# Detection of epigenetic field defects using a weighted epigenetic distance-based method

**DOI:** 10.1093/nar/gky882

**Published:** 2018-10-10

**Authors:** Ya Wang, Min Qian, Peifeng Ruan, Andrew E Teschendorff, Shuang Wang

**Affiliations:** 1 Department of Biostatistics, Mailman School of Public Health, Columbia University; 2 Department of Statistics, Columbian College of Arts and Sciences, the George Washington University; 3 CAS Key Lab of Computational Biology, Shanghai Institute for Biological Sciences, Chinese Academy of Sciences; 4 Statistical Cancer Genomics, UCL Cancer Institute, University College London

## Abstract

Identifying epigenetic field defects, notably early DNA methylation alterations, is important for early cancer detection. Research has suggested these early methylation alterations are infrequent across samples and identifiable as outlier samples. Here we developed a weighted epigenetic distance-based method characterizing (dis)similarity in methylation measures at multiple CpGs in a gene or a genetic region between pairwise samples, with weights to up-weight signal CpGs and down-weight noise CpGs. Using distance-based approaches, weak signals that might be filtered out in a CpG site-level analysis could be accumulated and therefore boost the overall study power. In constructing epigenetic distances, we considered both differential methylation (DM) and differential variability (DV) signals. We demonstrated the superior performance of the proposed weighted epigenetic distance-based method over non-weighted versions and site-level EWAS (epigenome-wide association studies) methods in simulation studies. Application to breast cancer methylation data from Gene Expression Omnibus (GEO) comparing normal-adjacent tissue to tumor of breast cancer patients and normal tissue of independent age-matched cancer-free women identified novel epigenetic field defects that were missed by EWAS methods, when majority were previously reported to be associated with breast cancer and were confirmed the progression to breast cancer. We further replicated some of the identified epigenetic field defects.

## INTRODUCTION

Identifying molecular alterations that happen early in carcinogenesis, known as field defects, is important for early cancer detection. One common approach is to compare normal tissue of healthy individuals to normal tissue adjacent to tumor (normal-adjacent tissue) of cancer patients as a surrogate of pre-cancer tissue that are difficult to collect. There have been studies in identifying epigenetic field defects (1–4), notably early DNA methylation alterations. DNA methylation is an epigenetic modification that has been shown to be crucial in gene expression (5–8) and cancers (9–12). There are mainly two types of aberrant DNA methylation in cancers, local hyper-methylation in promoter-related CpGs that leads to the silencing of downstream tumor suppressor genes (13–17), and global hypo-methylation that leads to chromosome instability (17–20). Studies have successfully identified epigenetic field defects in breast cancer by comparing normal-adjacent tissue of breast cancer patients to normal tissue from healthy individuals. Teschendorff *et al.* identified epigenetic field defects in breast cancer based on differential variability (DV), i.e. variance signals in DNA methylation (3), using methylation site-level analyses. Our previous work (21) identified epigenetic field defects in breast cancer based on both differential methylation (DM), i.e. mean signals, and DV, using methylation region-level analyses. In both studies, epigenetic field defects were found to be mainly driven by increased variation in methylation due to several outlier normal-adjacent tissue samples.

Due to the fact that CpG site-level signals for epigenetic field defects may be very small, existing methods based on differences (DM or DV or both) on CpG site-level may not have good power. Standard epigenome-wide association studies (EWAS) that focus on mean signals (EWAS-DM) perform CpG site-level tests to identify differentially methylated CpGs between two experimental groups using standard tests such as a t-test, a regression-based test or its regularized versions (22–24), or a non-parametric Wilcoxon rank sum test (25). EWAS that focus on variance signals (EWAS-DV) perform CpG site-level tests to identify differential variation CpGs between two experimental groups using standard tests such as the F-test (26,27), the Bartlett's test or its regularized version (3,4), or an empirical Bayes extension of the Levene's test (28). The F-test and Bartlett's test are sensitive to departures from normality which is usually the case for methylation data, while the Levene's test is more robust to non-normality. On the other hand, distance-based methods that characterize (dis)similarity between pairwise samples across a gene, a genetic region, a pathway or an entire genome have been proven to be powerful in genetic and gene expression studies (29–33). While standard EWAS perform CpG site-level tests with stringent multiple comparisons adjustment, in a gene or a genetic region level, the common practice using non-distance-based methods is to select the minimum *P*-value out of all CpGs in that region. These methods will not be powerful when site-level effects are very small. Alternatively, the distance-based methods accumulate any CpG site-level signals from a gene or a genetic region via the (dis)similarity matrix thus boost the overall association power, making them the ideal methods for detection of epigenetic field defects.

Here, we developed a weighted epigenetic distance-based method to identify epigenetic field defects at gene or genetic-region levels using both DM and DV signals. CpG site-level weights were incorporated in the calculation of (dis)similarity matrix to further boost signals and reduce noises. Specifically, we used original DNA methylation measures to examine DM and centered quadratic methylation measures to examine DV and considered site-level weights based on strengths of site-level DM and DV signals. Simulation studies showed much improved performance of the proposed weighted epigenetic distance-based method over several comparing methods including non-weighted versions and methods that use either DM or DV signals as well as standard EWAS methods. We further demonstrated the performance of the proposed method through an application to the 450K DNA methylation data of normal-adjacent tissue of breast invasive carcinoma (BRCA) patients and normal tissue from independent age-matched cancer-free women from Gene Expression Omnibus (GEO). The proposed method that accumulates weighted DM and DV signals identified genes with epigenetic field defects that were missed by standard EWAS methods and non-weighted distance-based methods. Many of these epigenetic field defects were previously reported to be associated with breast cancer. Further examination confirmed their enrichment in the progression to breast cancer and replicated some of these identified epigenetic field defects.

## MATERIALS AND METHODS

Case-control designs using normal tissue from healthy individuals ($Y = 0$) and normal tissue adjacent to tumor from cancer patients ($Y = 1$) as a surrogate of pre-cancer tissue are widely used to identify epigenetic field defects in cancers. We therefore focused on case-control designs and illustrated and applied the proposed weighted epigenetic distance-based method on gene level. However, the proposed method can be easily adapted to other types of design and on genetic region or genome levels. There are three steps in the proposed distance-based method: (i) to define gene-level weighted epigenetic distance matrix; (ii) to calculate pseudo-$F$statistic and (iii) to assess statistical significance using permutations.

### Step 1: Define gene-level weighted epigenetic distance matrix

#### Define epigenetic distance matrix

For each gene, let ${{{\bf X}}^m}$ be an $2N \times n$ matrix with original DNA methylation measures for $N$ cases and $N$ controls of $n$ CpG sites in a gene, where element $x_{ij}^m$ harbors DNA methylation measure of the $j$th CpG site, $j = 1,...,n$ in the gene, for the $i$-th subject, $i = 1,...,N$. This ${{{\bf X}}^m}$ matrix will be used to examine differential methylation (DM) capturing methylation mean signals. Let ${{{\bf X}}^v}$ be an $2N \times n$ pseudo data matrix of variability score capturing methylation variance signals, which will be used to examine differential variability (DV). The element $x_{ij}^v = {( {x_{ij}^m - \bar{x}_j^m} )^2}$ harbors centered quadratic methylation measure of the same $j$th CpG site for the $i$th subject. Here $\bar{x}_j^m = \frac{1}{N}\sum\nolimits_{i = 1}^N {x_{ij}^m}$ is the mean methylation measure of the $j$-th CpG site across $N$ cases and $N$ controls separately. The quadratic terms are centered to better capture variance signals. By using ${{{\bf X}}^{mv}} = [ {{{{\bf X}}^m},{{{\bf X}}^v}} ]$, an $2N \times 2n$ matrix, we will be able to capture both methylation mean and methylation variance signals of the $n$ CpG sites. Before constructing the epigenetic distance between any pair of subjects, we performed normalization on each column of ${{{\bf X}}^{mv}}$ such that each column has mean zero and unit standard deviation.

We define the $2N \times 2N$ epigenetic distance matrix ${{{\bf D}}^{DM - DV}}$ with element $d_{st}^{DM - DV}$ that captures dissimilarities between any given pair of individuals $s$ and $t$, $s,t = 1,...,2N$ as

(1) \begin{equation*}d_{st}^{DM - DV} = \sqrt {\sum\limits_{j = 1}^n {\left\{ {\frac{1}{{2n}}{{\left( {x_{sj}^m - x_{tj}^m} \right)}^2} + \frac{1}{{2n}}{{\left( {x_{sj}^v - x_{tj}^v} \right)}^2}} \right\}} } .\end{equation*}

#### Incorporate CpG site-level weights into epigenetic distance matrix

We construct CpG site-level weights aiming to up-weight signal CpGs (mean or variance) and to down-weight noise CpGs in calculating distances between pairs of subjects. Therefore, we define weights for mean and variance signals at CpG site $j$ as follows:

(2) \begin{equation*}w_j^m = \frac{{ - {{\log }_{10}}(p_j^m)}}{{\sum\nolimits_{j = 1}^n { - {{\log }_{10}}(p_j^m)} }},\quad w_j^v = \frac{{ - {{\log }_{10}}(p_j^v)}}{{\sum\nolimits_{j = 1}^n { - {{\log }_{10}}(p_j^v)} }}\end{equation*}

where $p_j^m$ and $p_j^v$ are the *P*-values from the two-sided two-sample $t$-test testing if the mean methylation measures are the same between cases and controls and from the one-sided Levene's test testing if the variance of the methylation measures in cases is greater than that in controls at CpG site $j$, $j = 1,...,n$ in a gene. Note that $\sum\nolimits_{j = 1}^n {w_j^m = } \sum\nolimits_{j = 1}^n {w_j^v = } 1$.

The corresponding $2N \times 2N$ weighted epigenetic distance matrix ${{{\bf D}}^{w - DM - DV}}$ with element $d_{st}^{w - DM - DV}$ that captures weighted dissimilarities between individuals $s$ and $t$, $s,t = 1,...,2N$ can be defined as

(3) \begin{equation*}d_{st}^{w - DM - DV} = \sqrt {\sum\limits_{j = 1}^n {\left\{ {\frac{{w_j^m}}{2}{{\left( {x_{sj}^m - x_{tj}^m} \right)}^2} + \frac{{w_j^v}}{2}{{\left( {x_{sj}^v - x_{tj}^v} \right)}^2}} \right\}} } .\end{equation*}

### Step 2: Calculate pseudo-*F* statistic

We apply distance-based regression originally developed in the field of ecology (31,32) to test if DNA methylation measures in a gene is associated with the case-control status. Specifically, for each gene, we calculate a pseudo-$F$ statistic based on the weighted epigenetic distance matrix ${{{\bf D}}^{w - DM - DV}}$ introduced above

(4) \begin{equation*}{F^{w - DM - DV}} = \frac{{tr({{\bf HGH}})}}{{tr[({{\bf I}} - {{\bf H}}){{\bf G}}({{\bf I}} - {{\bf H}})]}}\end{equation*}

where ${{\bf H}} = {{\bf Y}}{({{{\bf Y}}^T}{{\bf Y}})^{ - 1}}{{{\bf Y}}^T}$ is an $2N \times 2N$ projection matrix, $Y$ is an $2N \times 1$ vector with case ($Y = 1$) and control ($Y = 0$) status, ${{\bf G}} = \left({{\bf I}} - \frac{1}{{2N}}{{\bf 1}}{{{\bf 1}}^T} \right){{\bf A}} \left({{\bf I}} - \frac{1}{{2N}}{{\bf 1}}{{{\bf 1}}^T} \right)$ is the Gower's centered matrix, ${{\bf A}} = ({a_{st}}) = \left({ - \frac{1}{2}{{( {d_{st}^{w - DM - DV}} )}^2}} \right)$, ${{\bf 1}}$ is an $2N$-dimensional column vector with elements 1, and ${{\bf I}}$ is an $2N \times 2N$ identity matrix. The pseudo-$F$ statistic is used to evaluate the association between epigenetic similarity of a gene with n CpG sites and the case/control status.

### Step 3: Assess statistical significance using permutations

To access significance of all $G$ genes tested, we use permutation procedures, where we randomly shuffle cases ($Y = 1$) and controls ($Y = 0$) and repeat Steps 1–2 on the permuted data. In order to have more granular *P*-values, we pool pseudo-$F$ statistics of all $G$ genes from all permutations, as well as those from the observed data, to compute the empirical *P*-value (34). We repeat the permutation procedure 999 times, and calculate the empirical *P*-value for gene $g$, $g = 1,...,G$, as follows:

(5) \begin{equation*}P_g^{w - DM - DV} = \frac{{\sum\nolimits_{g^\prime = 1}^G {\left\{ {1 + \sum\nolimits_{{\rm{perm}} = 1}^{999} {I\left( {F_{g^\prime,{\rm{perm}}}^{w - DM - DV} \ge F_g^{w - DM - DV}} \right)} } \right\}} }}{{G \times (1 + 999)}}\end{equation*}

In the real data application, we have $G$= 19 271 genes, which helps to have high resolution gene-level empirical *P*-values.

### Comparing methods

We compare the performance of the proposed method ${{{\bf D}}^{w - DM - DV}}$ that considers site-level weights for mean and variance signals to that of several comparing methods, including the weighted distance-based methods that consider mean signals only ${{{\bf D}}^{w - DM}}$ or variance signals only ${{{\bf D}}^{w - DV}}$, and distance-based methods without weights that consider both mean and variance signals ${{{\bf D}}^{DM - DV}}$, mean signals only ${{{\bf D}}^{DM}}$, variance signals only ${{{\bf D}}^{DV}}$, and standard EWAS methods on each CpG site with multiple comparisons adjustment of number of CpGs in a gene based on mean signals $EWA{S^{DM}}$ or variance signals $EWA{S^{DV}}$.

### Simulation study

We conducted simulation studies to evaluate type I error rate and power of the proposed method ${{{\bf D}}^{w - DM - DV}}$ and those of the comparing methods described above. Type I error rate is defined as the proportion of simulations with any significant genes when the data is generated under the null hypothesis of no genes are associated with case-control status. Power is defined as the proportion of simulations with observed pseudo-$F$ statistics smaller than that of the permuted values from all genes across all permutations.

### Simulation setup

We simulated methylation measures $X$ of cases ($Y = 1$) and controls ($Y = 0$) at every CpG site in a gene from beta distributions:

\begin{equation*}X|Y = 0 \sim {\rm{Beta}}({a_0},{b_0})\end{equation*}

\begin{equation*}X|Y = 1 \sim {\rm{Beta}}({a_1},{b_1})\end{equation*}

where shape parameters ${a_0}$ and ${b_0}$ for samples in the control group were chosen based on estimates from the 50 normal tissue samples from cancer-free women in the GEO BRCA data (GSE69914), and shape parameters ${a_1}$ and ${b_1}$ for samples in the case group were chosen based on estimates from the 42 normal-adjacent tissues in the GEO BRCA data. More specifically, the average of the methylation means and standard deviations (SDs) of all CpG sites with gene information for the 50 normal tissue samples is 0.47 and 0.05, respectively. Therefore, we set ${a_0} = 46.36$ and ${b_0} = 52.28$ for noise CpGs such that the corresponding mean and SD of the beta distribution are 0.47 and 0.05, respectively. We generated methylation measures for 40 cases and 40 controls to mimic the size of the GEO BRCA study. We set ${a_1} = {a_0}$ and ${b_1} = {b_0}$ for all CpG sites in case and control groups to evaluate type I error rates. For power scenarios, we considered scenarios when signal CpGs have different mean or variance signals through varying shape parameters ${a_1}$ and ${b_1}$. We conducted 1000 simulations in each simulation setting.

#### Simulation settings with one gene

We first considered one gene with different number of CpGs with different signal-to-noise ratios of the CpGs. That is, the ratio between number of signal CpGs and number of noise CpGs in this gene ranges from 1:0, 1:24, 1:49, 3:47, to 5:45. We considered scenarios when signal CpGs have different mean or variance signals by varying shape parameters ${a_1}$ and ${b_1}$ such that the mean differences in methylation measures between cases and controls are 0.02, 0.04 0.06, 0.08 and 0.1, and the ratios of SDs for cases and controls are 1.25, 1.50, 1.75, 2, 2.25 and 2.50, respectively. The values of ${a_1}$, ${b_1}$ in those scenarios and the corresponding effect sizes are summarized in the Supplementary Table S1. We consider a gene to be significant at the 0.05 significance level.

#### Simulation settings with 10 genes

We then considered 10 genes with one gene having signals when there are 25 CpGs in each of the 10 genes. In the signal gene, we set one CpG to have mean or/and variance signals with different effect sizes. Here we test for the global null and consider a simulation study to be significant if any gene is significant after Bonferroni adjustment for testing 10 genes. The empirical *P*-value for each gene is calculated using formula 5, where *G* = 10.

#### Simulation settings with outliers

Since epigenetic field defects are often characterized by increased variation in DNA methylation due to a few outlier normal-adjacent tissue samples (3,21), we considered simulation scenarios with outlier samples. Here, we only considered one gene with 50 CpGs for illustration purposes. We considered two signal-to-noise ratios in this gene to be either 5:45 or 10:40. We set 10%, 15% or 20% of cases to be outlier samples with DNA methylation alterations at some signal CpGs, while the rest cases have the same methylation measures as controls at those signal CpGs when different outlier samples could have DNA methylation alterations at different signal CpGs, For each signal CpG, we generated methylation measures $X$ for cases from a mixture distribution $X = (1 - Z){X_1} + Z{X_2}$, and methylation measures for controls from ${X_1} \sim {\rm{Beta}}({a_0},{b_0})$. Specifically, at each signal CpG, we randomly assigned 40 cases to be either outlier samples ($Z = 1$) or non-outlier samples ($Z = 0$) by $Z \sim {\rm{Bernoulli}}(p)$, where $p$ is the probability of any case being an outlier sample. We then generated methylation measures of outlier samples from ${X_2} \sim {\rm{Beta}}({a_2},{b_2})$ and non-outlier samples from ${X_1} \sim {\rm{Beta}}({a_0},{b_0})$.

#### Simulation settings with one gene considering correlations among CpGs

Since neighboring CpGs are known to be correlated, we considered simulation scenarios that assume an AR(1) correlation among CpGs in a gene with a correlation coefficient 0.5. The detailed information for simulation setup for this scenario is summarized in the Supplementary File section 2 Simulation settings with one gene considering correlations among CpGs.

## RESULTS

### Simulation results

#### Type I error rate

Type I error rates are well controlled at the 0.05 significance level in settings with one gene and 10 genes after Bonferroni adjustment for multiple comparisons (Table 1), respectively.

**Table 1.** Type I error rates

	1 gene			10 genes^a^
Methods	1 CpG^b^	25 CpGs	50 CpGs	25 CpGs
${{{\bf D}}^{w - DM - DV}}$	0.044	0.044	0.037	0.050
${{{\bf D}}^{w - DM}}$	0.046	0.032	0.048	0.053
${{{\bf D}}^{w - DV}}$	0.048	0.056	0.048	0.049
${{{\bf D}}^{DM - DV}}$	0.044	0.052	0.045	0.054
${{{\bf D}}^{DM}}$	0.046	0.043	0.041	0.057
${{{\bf D}}^{DV}}$	0.044	0.052	0.054	0.045
$EWA{S^{DM}}$	0.046	0.030	0.039	0.050
$EWA{S^{DV}}$	0.044	0.047	0.040	0.037

^a^Type I error rates after Bonferroni adjustment for 10 genes.

^b^Number of CpG sites in a gene.

#### Power for simulation settings with one gene

**Figure 1.**
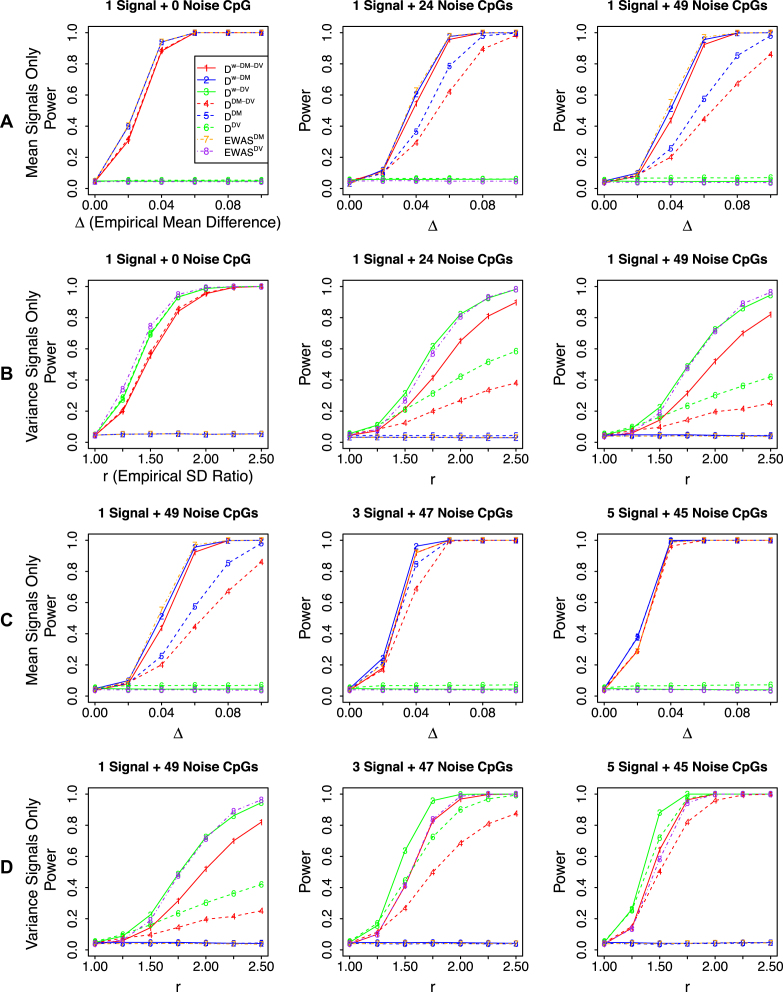
Power results for simulation settings with one gene. The signal gene has one signal CpG and increasing number of total CpGs, i.e., decreasing signal-to-noise ratios from 1:0, 1:24 to 1:49 (panel **A** for mean signals only, panel **B** for variance signals only), or with a fixed total number of CpGs 50 and increasing signal-to-noise ratios from 1:49, 3:47, to 5:45 (panel **C** for mean signals only, panel **D** for variance signals only).

Power results for simulation settings with one gene are summarized in Figure 1. When there are only mean signals at signal CpGs, ${{{\bf D}}^{w - DV}}$, ${{{\bf D}}^{DV}}$ and $EWA{S^{DV}}$ that consider variance signals only do not have any power as expected. When there is only one CpG in the gene, the non-weighted distance-based methods are the same as the weighted versions, as well as the EWAS method as expected. When there is one signal CpG and increasing number of noise CpGs in the gene, power of ${{{\bf D}}^{DM}}$ decreases drastically while power of the weighted version ${{{\bf D}}^{w - DM}}$ are well maintained. This suggests that incorporating weights to CpGs indeed helps to up-weight signal CpGs and down-weight noise CpGs in constructing the distance matrix, thus improves the performance. When the size of a gene, i.e., number of CpGs in a gene, is fixed, among which when the number of signal CpGs increases, power of ${{{\bf D}}^{w - DM}}$ increases much slower than that of ${{{\bf D}}^{DM}}$ while ${{{\bf D}}^{w - DM}}$ always has greater power than that of ${{{\bf D}}^{DM}}$. This implies that adding weights is most effective when a small percent of CpGs in a gene are signals. Similar power patterns are observed between weighted and non-weighted versions of the distance-based methods that consider both mean and variance signals, ${{{\bf D}}^{w - DM - DV}}$ and ${{{\bf D}}^{DM - DV}}$. We also notice that ${{{\bf D}}^{w - DM - DV}}$ is slightly less powerful than ${{{\bf D}}^{w - DM}}$ because the overall mean signals are diluted by the inclusion of pseudo-sites for variance when there are only mean signals in the data. Moreover, ${{{\bf D}}^{w - DM}}$ slightly outperform $EWA{S^{DM}}$ when there are several signal CpGs. This is because the distance-based method has the advantage to accumulate weak signals and thus boost the overall power.

Similar power patterns are observed when signal CpGs are set to have variance signals only. ${{{\bf D}}^{w - DM}}$, ${{{\bf D}}^{DM}}$ and $EWA{S^{DM}}$ that consider mean signals only do not have any power, and the weighted distance-based methods outperform the non-weighted versions in the presence of noise CpGs, and ${{{\bf D}}^{w - DV}}$ performs better than ${{{\bf D}}^{w - DM - DV}}$, and ${{{\bf D}}^{w - DV}}$ outperforms $EWA{S^{DV}}$ when there are several signal CpGs.

#### Power for simulation settings with 10 genes

**Figure 2.**
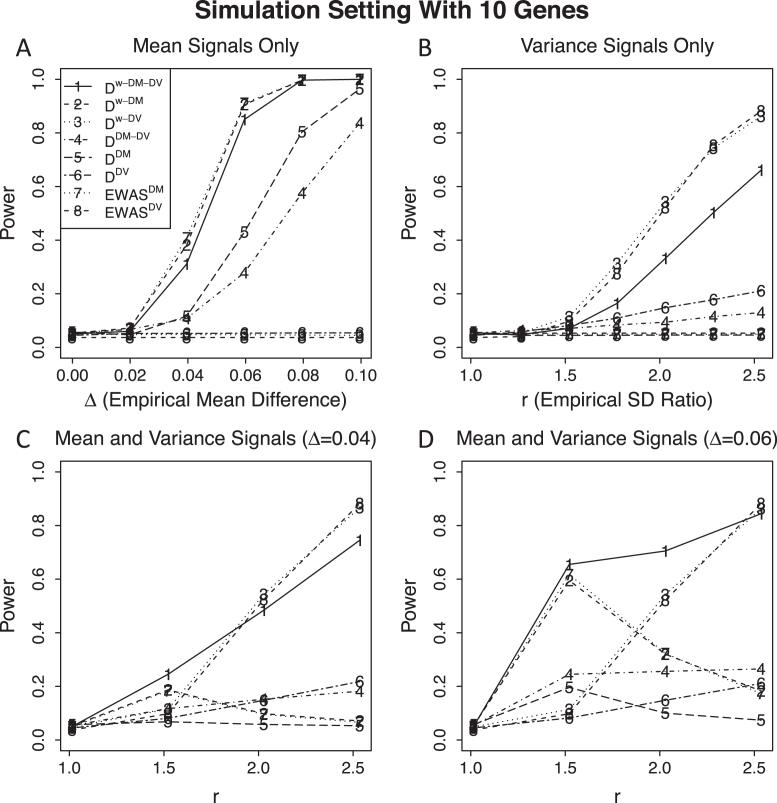
Power results for simulation settings with 10 genes. We set each gene to have 25 CpGs and only one gene to have signals. The signal gene has 1 signal CpG and 24 noise CpGs, with signal CpG having mean signal only (panel **A**), variance signal only (panel **B**), and mean and variance signals with different sizes of mean signals (panels **C** and **D**).

Power results for simulation settings with 10 genes are summarized in Figure 2. When signal CpGs have either mean or variance signals, we observed similar patterns as in the simulation settings with one gene. When signal CpGs have non-negligible mean signals and variance signals ranging from weak to strong, ${{{\bf D}}^{w - DM - DV}}$ performs the best when variance signals are also weak to moderate as expected. This confirms that the potential area of usage for distance-based methods to be most effective is when there are weak signals that could be accumulated to boost the study power. When there are very strong signals at some sites, any methods will perform well. One observation that we need to point out is, powers of ${{{\bf D}}^{w - DM}}$, ${{{\bf D}}^{DM}}$ and $EWA{S^{DM}}$ that only consider mean signals actually decrease as variance signals increase when mean signals exist. This is due to the fact that we worked on the standardized data in ${{{\bf X}}^{mv}} = [ {{{{\bf X}}^m},{{{\bf X}}^v}} ]$, and the effect sizes of mean signals (standardized mean difference) decrease as the effect sizes of variance signals (ratio of standard deviation for cases and controls) increase after standardization.

#### Power for simulation settings with outliers

**Figure 3.**
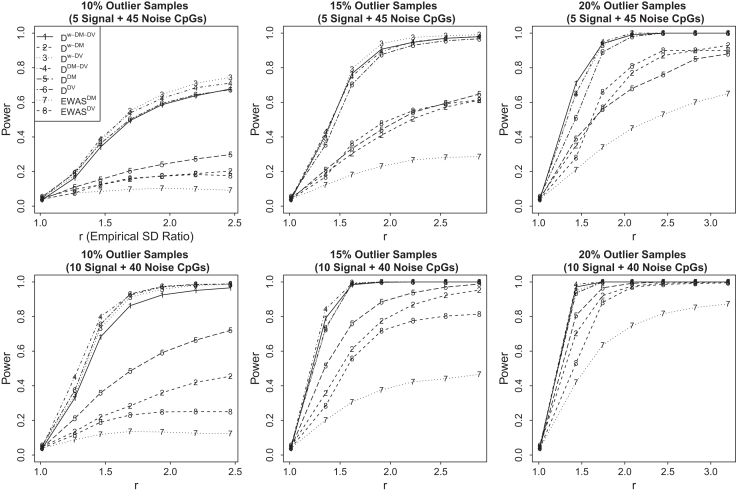
Power results for simulation settings with outlier samples. We set to have 10%, 15% and 20% outlier samples and two different signal-to-noise ratios 5:45 and 10:40.

Power results for simulation settings with outlier samples are summarized in Figure 3. We observe that power of all methods increases as the signal-to-noise ratio increases and as the proportion of outlier samples increases as expected, and distance-based methods outperform non-distance-based methods while $EWA{S^{DM}}$ and $EWA{S^{DV}}$ have very little power when there are only 10% outlier samples. Among distance-based methods, ${{{\bf D}}^{w - DM}}$ and ${{{\bf D}}^{DM}}$ that consider mean signals only have lower power compare to other methods as the mean signals introduced by a few outlier samples are usually too weak to be detected by methods that consider mean signals only. On the other hand, ${{{\bf D}}^{DM - DV}}$ that considers both mean and variance signals outperforms methods that consider variance signals only, ${{{\bf D}}^{DV}}$.The two weighted distance-based methods ${{{\bf D}}^{w - DM - DV}}$ and ${{{\bf D}}^{w - DV}}$ are among the best performed methods consistently. This implies the superiority of ${{{\bf D}}^{w - DM - DV}}$ in the presence of weak signals in both DM and DV.

#### Power for simulation settings with one gene considering correlations among CpGs

The type I error rates under this scenario are summarized in Supplementary Table S2. The power results are summarized in Supplementary Figure S1. We note that the power patterns are very similar to those observed in simulations ignoring correlations among CpG sites. This implies that the correlations among neighboring CpGs do not have much impact on the performance of the proposed distance-based methods.

### Real data application

We applied the proposed method ${{{\bf D}}^{w - DM - DV}}$ and all the comparing methods to two GEO 450K DNA methylation data of breast invasive carcinoma (BRCA) (GSE69914 and GSE67919). As we have demonstrated the superior power of ${{{\bf D}}^{w - DM - DV}}$ over other distance-based methods in the simulation studies, we focused on ${{{\bf D}}^{w - DM - DV}}$ in the real data application and compared its performance to that of the EWAS method in the main text and included results using all other comparing distance-based methods in the Supplementary File section 3 Real data application.

In order for the two EWAS methods, $EWA{S^{DM}}$ and $EWA{S^{DV}}$, to have a fair comparison with ${{{\bf D}}^{w - DM - DV}}$, we first adjusted multiple comparisons for the number of CpGs in a gene by multiplying the site-level *P*-values based on DM and DV with the number of CpGs in the gene, and then selected the minimum adjusted DM and DV *P*-value across all *P*-values in the gene as the gene-level *P*-value. We refer to this method as $EWA{S^{\min - P}}$.

### Discovery analysis using the GEO BRCA data

We applied the proposed method ${{{\bf D}}^{w - DM - DV}}$ and the comparing methods to the GEO 450K DNA methylation data of normal-adjacent tissue of breast invasive carcinoma (BRCA) patients and normal tissue from independent age-matched cancer-free women (GSE69914). In the original GEO BRCA data, there are DNA methylation measures on 485,512 CpGs for 42 tumor and normal-adjacent pairs from breast cancer patients, 50 normal tissue of independent age-matched cancer-free women and 263 additional tumor tissue of independent breast cancer patients. We conducted standard quality control steps where we removed CpGs on sex chromosomes and those contain either a SNP at the CpG interrogation or at the single nucleotide extension (SBE) based on UCSC dbSNP table version 147 using the R package ‘IlluminaHumanMethylation450kanno.ilmn12.hg19’ (35). We also required at least 95% CpG coverage per sample and 70% sample coverage per CpG, and only kept CpGs with gene annotations. We ended up with 344 947 CpGs, covering 19 271 genes, from 42 normal-adjacent tissues, 50 normal tissues and 263 independent tumor tissues.

**Figure 4.**
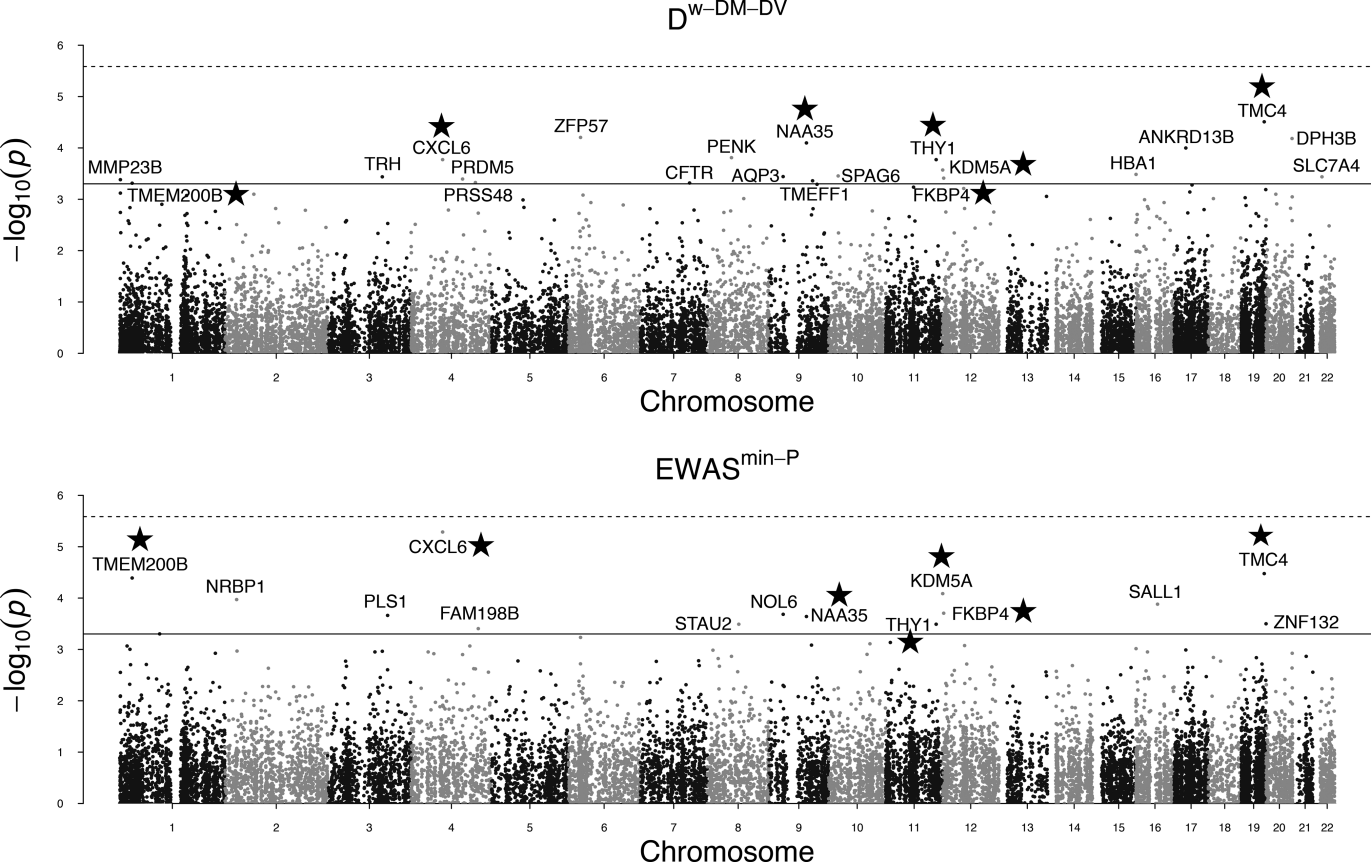
Manhattan plots with results from ${{{\bf D}}^{w - DM - DV}}$ and $EWA{S^{\min - P}}$. The solid horizontal line is the 0.0005 gene-level *P*-value threshold. The dashed horizontal line is the Bonferroni adjusted 0.05 significance level (0.05/19 271 genes = 0.0000026 adjusted gene-level *P*-value threshold). Genes annotated with stars are genes identified by both methods at the 0.0005 gene-level *P*-value threshold.

Since Bonferroni adjustment for multiple comparisons of the 19 271 genes is too conservative, especially with the small sample size in the GEO BRCA dataset, we used a less stringent threshold 0.0005 on empirical gene-level *P*-values obtained from the permutation procedure (Figure 4). Our main purpose is to demonstrate the superior performance of the proposed method ${{{\bf D}}^{w - DM - DV}}$ over several comparing methods, especially the EWAS methods. Results using ${{{\bf D}}^{w - DM - DV}}$ and $EWA{S^{\min - P}}$ comparing 42 normal-adjacent tissues to 50 normal tissues are shown in the Manhattan plots in Figure 4. At the 0.0005 threshold for gene-level *P*-values, ${{{\bf D}}^{w - DM - DV}}$ identified 21 genes (Table 2), of which 18 were previously reported to be associated with breast cancer; $EWA{S^{\min - P}}$ identified 14 genes (Table 3), of which 9 were previously reported to be associated with breast cancer. There are 7 overlapping genes, *TMC4, NAA35, THY1, CXCL6, KDM5A, FKBP4*, and *TMEM200B* that were identified by both methods. Except for the *PLS1* gene, the 7 genes uniquely identified by $EWA{S^{\min - P}}$ all rank very high in ${{{\bf D}}^{w - DM - DV}}$ results out of the 19 271 genes (Table 3). Except for the *CFTR* gene, the 14 genes uniquely identified by ${{{\bf D}}^{w - DM - DV}}$ also all rank very high in $EWA{S^{\min - P}}$ results. This suggests an overall good consistency between results of ${{{\bf D}}^{w - DM - DV}}$ and $EWA{S^{\min - P}}$. At the same 0.0005 gene-level *P*-value threshold, other comparing methods ${{{\bf D}}^{w - DM}}$, ${{{\bf D}}^{w - DV}}$, ${{{\bf D}}^{DM - DV}}$, ${{{\bf D}}^{DM}}$ and ${{{\bf D}}^{DV}}$ identified 11, 9, 2, 6 and 4 genes, of which 6, 7, 1, 3 and 1 genes were also identified by the proposed ${{{\bf D}}^{w - DM - DV}}$ (Supplementary Tables S3–S7), respectively.

**Table 2.** Twenty one genes identified by ${{{\bf D}}^{w - DM - DV}}$ at the 0.0005 gene-level *P*-value threshold using the GEO BRCA Data

Rank	Gene	# CpG	Cancer	Rank in $EWA{S^{\min - P}}$
1	TMC4*	13	Breast Cancer (37)	2
2	*ZFP57*	5	Breast Cancer (38)	16
3	*DPH3B*	5	–	61
4	NAA35*	7	Breast Cancer (39)	10
5	*ANKRD13B*	22	Breast Cancer (40)	25
6	*PENK*	23	Breast Cancer (41)	37
7	THY1*	19	Breast Cancer (42)	13
8	CXCL6*	7	Breast Cancer (43)	1
9	KDM5A*	2	Breast Cancer (44)	4
10	*HBA1*	7	Breast Cancer (45)	23
11	*SPAG6*	16	Acute Myeloid Leukemia (46)	170
12	*AQP3*	7	Breast Cancer (47)	140
13	*TRH*	16	Breast Cancer (48)	28
14	*SLC7A4*	12	Breast Cancer (49)	175
15	FKBP4*	18	Breast Cancer (50)	7
16	*PRDM5*	18	Breast Cancer (51)	36
17	*MMP23B*	2	Breast Cancer (52)	80
18	*TMEFF1*	5	Breast Cancer (53)	156
19	*PRSS48*	7	–	64
20	*CFTR*	16	Breast Cancer (54)	1055
21	TMEM200B*	20	Breast Cancer (55)	3

*Genes identified by both ${{{\bf D}}^{w - DM - DV}}$ and $EWA{S^{\min - P}}$.

**Table 3.** Fourteen genes identified by $EWA{S^{\min - P}}$ at the 0.0005 gene-level *P*-value threshold using the GEO BRCA Data

Rank	Gene	# CpG	Top CpG Signal^a^	Cancer	Rank in ${{{\bf D}}^{w - DM - DV}}$
1	CXCL6*	7	Variance	Breast Cancer (43)	11
2	TMC4*	13	Variance	Breast Cancer (37)	1
3	TMEM200B*	20	Variance	Acute Myeloid Leukemia (56)	41
4	KDM5A*	2	Variance	Breast Cancer (44)	4
5	*NRBP1*	12	Variance	Breast Cancer (57)	110
6	*SALL1*	44	Variance	Breast Cancer (58)	887
7	FKBP4*	18	Variance	Breast Cancer (50)	32
8	*NOL6*	5	Variance	-	160
9	*PLS1*	16	Variance	Bladder Cancer (59)	1069
10	NAA35*	7	Variance	Breast Cancer (39)	6
11	*ZNF132*	12	Mean	Prostate Cancer (60)	118
12	*STAU2*	39	Variance	Hepatocellular Carcinoma (61)	666
13	THY1*	19	Variance	Breast Cancer (42)	14
14	*FAM198B*	14	Variance	Breast Cancer (62)	84

^a^Mean or variance tests with smaller *P*-value at the most significant CpG in a gene.

*Genes identified by both ${{{\bf D}}^{w - DM - DV}}$ and $EWA{S^{\min - P}}$.

We further examined the 14 and 7 genes uniquely identified by ${{{\bf D}}^{w - DM - DV}}$ and $EWA{S^{\min - P}}$, respectively. We plotted heatmaps of the original DNA methylation measures of CpG sites on these genes for the 50 normal tissues, 42 normal-adjacent tissues together with the 42 matched tumor tissues (Supplementary Figures S2 and S3). In general, the 14 genes uniquely identified by ${{{\bf D}}^{w - DM - DV}}$ are genes with multiple CpGs of weak signals, i.e. weak dense signals. Moreover, some of these weak dense signals were mainly due to a few outlier normal-adjacent tissue samples, thus were missed by $EWA{S^{\min - P}}$. The seven genes uniquely identified by $EWA{S^{\min - P}}$ are those with just one or two CpGs with very strong signals, i.e. strong sparse signals. We also plotted heatmaps of seven genes identified by both ${{{\bf D}}^{w - DM - DV}}$ and $EWA{S^{\min - P}}$ (Supplementary Figure S4).

**Figure 5.**
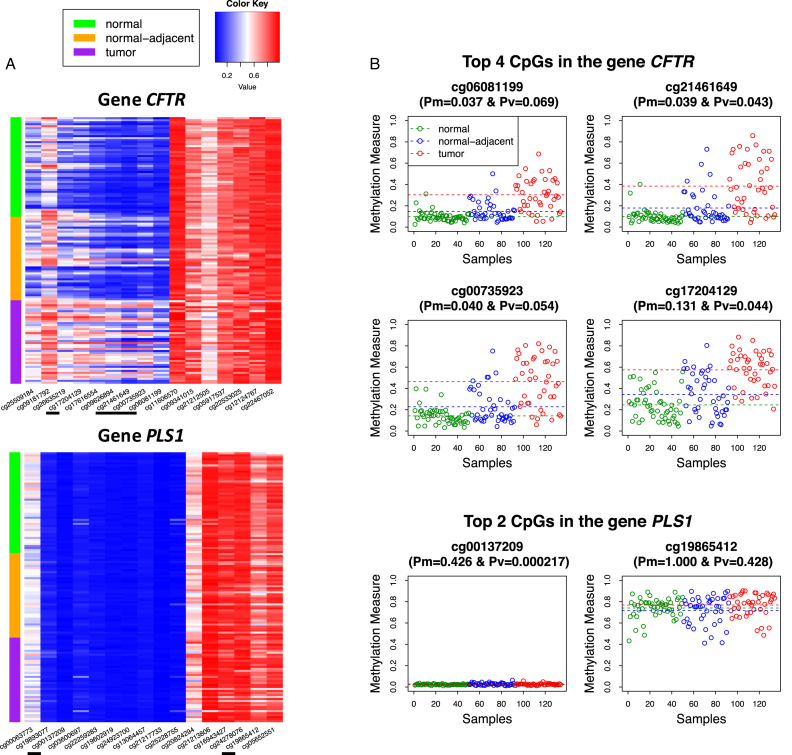
(**A**) Heatmaps of DNA methylation measures of CpGs in the *CFTR* and *PLS1* genes. The CpGs underlined are the top 4 *P*-value ranked CpGs in the *CFTR* gene and the top 2 *P*-value ranked CpGs in the *PLS1* gene. (**B**) DNA methylation measures of 50 normal tissues, 42 normal-adjacent tissues and 42 matched tumors of the top 4 *P*-value ranked CpGs in the *CFTR* gene and the top 2 *P*-value ranked CpGs in the *PLS1* gene. Pm and Pv are *P*-values from CpG site-level mean and variance tests adjusted for multiple comparisons for the number of CpGs in the gene. The three horizontal lines represent mean methylation levels of the three groups of normal tissues, normal-adjacent tissues and matched tumors.

We then investigated the two genes, *CFTR* and *PLS1*, that were uniquely identified by ${{{\bf D}}^{w - DM - DV}}$ and $EWA{S^{\min - P}}$, respectively, but ranked the last using the other method among all uniquely identified genes. We similarly plotted the heatmap of the original DNA methylation measures of CpG sites in these two genes (Figure 5A). For the *CFTR* gene that has 16 CpGs, it is clear that variation in methylation measures increases in the progression from normal tissues to normal-adjacent tissues and to tumor tissues in multiple CpGs when there are several samples among the 42 normal-adjacent tissue samples that are very different from the normal samples. On the other hand, for the *PLS1* gene that also has 16 CpGs, it was identified uniquely by $EWA{S^{\min - P}}$ because of one signal CpG site cg00137209 (Figure 5A), mainly due to the very small variation in the methylation measures of the normal tissues. We then plotted DNA methylation measures of the top 4 *P*-value ranked CpGs, ranked by CpG site-level *P*-values from both mean and variance tests each after adjusting for multiple comparisons for the number of CpGs in the *CFTR* gene (Figure 5B), which clearly shows elevated methylation levels in the progression to tumor. For the *PLS1* gene, we similarly plotted the DNA methylation measures of the top 2 *P*-value ranked CpGs (Figure 5B), where the #1 ranked CpG cg00137209 is the one that shows strong variance signal due to very small variation in the methylation measures of the normal tissues, when neither CpGs showed any enrichment in methylation measures in the progression to tumor. This suggests that genes uniquely identified by $EWA{S^{\min - P}}$ due to extreme *P*-values at one or two CpGs may not be reliable, while genes identified uniquely by ${{{\bf D}}^{w - DM - DV}}$ are generally characterized by multiple signal CpGs, thus are more reliable.

We also plotted the DNA methylation measures of all CpGs in these two genes *CFTR* and *PLS1* (Supplementary Figures S5 and S6, respectively). It is again clear that almost half of the CpGs in the *CFTR* gene have weak mean signals and weak variance signals, thus missed by $EWA{S^{\min - P}}$ due to stringent multiple comparisons adjustment. In addition, we plotted the weighted distance matrices of the 50 normal tissues and the 42 normal-adjacent tissues for the *CFTR* gene and the *PLS1* gene (Supplementary Figure S7). For the *CFTR* gene, we observe little variation in distances among normal samples and increased variation in distances between several pairs of normal and normal-adjacent samples, while for the *PLS1* gene, we observe no clear pattern. We also plotted the DNA methylation measures of CpGs in the *TMC4* gene (Supplementary Figure S8) that was identified by both ${{{\bf D}}^{w - DM - DV}}$ and $EWA{S^{\min - P}}$ and ranked #1 and #2 in the two methods, respectively. There are 13 CpGs in the *TMC4* gene, 3 CpGs have strong variance signals when two of the three CpGs also have mean signals. Thus, the *TMC4* gene was identified by both ${{{\bf D}}^{w - DM - DV}}$ and $EWA{S^{\min - P}}$.

In our previous work on differentially methylated regions (DMRs) using the same GEO BRCA data, we identified 2 DMRs of epigenetic field defects using both mean and variance signals (21). The two DMRs cover two genes, *NKX6−2* and *CCND2*, which rank #113 and #359 in the ${{{\bf D}}^{w - DM - DV}}$ results. Further investigation revealed that the two DMRs only cover part of the two genes. We therefore broke down the two genes into smaller parts so that there is one part that covers exactly the identified DMR. We then treated these smaller parts as individual regions and repeated ${{{\bf D}}^{w - DM - DV}}$ across the whole genome. The rank of the *NKX6−2* part that matches with the DMR moved up to #90 from #113 while the other two parts rank #107 and #4855, respectively. The rank of the *CCND2* part that matches with the other DMR moved up to #154 from #359 and the other part ranks #1116. Overall, the 2 DMR-covered genes previously identified as epigenetic field defects also rank on top in the results of ${{{\bf D}}^{w - DM - DV}}$. This suggests that we may combine DMR detection techniques with distance-based methods to first better define ‘regions of interest’ using DMR ideas and then assess significance more powerfully with distance-based methods.

We also investigated the relation between the number of CpGs in a gene and the probability that the gene is selected, where we binned genes based on their sizes and calculated the selection probability of a gene in a bin as the proportion of genes identified out of all genes in the bin. We plotted the selection probabilities against gene sizes (Supplementary Figure S9) and found that the selection probabilities for different versions of the distance-based methods and $EWA{S^{\min - P}}$ method are not systematically affected by gene sizes.

### Validation of the identified epigenetic field defects in the GEO BRCA data

We further validated the 21 genes of epigenetic field defects identified by ${{{\bf D}}^{w - DM - DV}}$ through comparing methylation measures of the 21-gene-covered CpGs between 263 independent tumor tissues and 42 normal-adjacent tissues to examine if the methylation levels at these CpGs exhibit progression to tumor. Specifically, we performed the two-sample $t$-test at each of these CpGs and plotted the $ - {\log _{10}}(p{\rm{ - value}})$ from the two comparisons, 50 normal tissues versus 42 normal-adjacent tissues and 42 normal-adjacent tissues vs. 263 tumor tissues (Supplementary Figure S10). In general, the majority of these CpGs show more significant signals in the progression from normal tissues to normal-adjacent tissues to tumor.

### Replication analysis using an independent data of normal tissues

As epigenetic field defects identified in one set of normal vs. normal-adjacent comparison maybe driven by a few ‘outlier’ normal-adjacent samples (3,4,21), different epigenetic field defects could be identified in a different set of normal versus normal-adjacent comparison that are driven by different ‘outlier’ normal-adjacent samples. Therefore, we propose to conduct a replication analysis that uses the same normal-adjacent tissue samples but compare to an independent data of normal samples. We used 450K DNA methylation data of 18 normal tissue of 18 breast reduction mammoplasty subjects (GSE67919) (36). The original data have methylation measures on 485 577 CpG sites. We followed the same quality control steps as for the discovery GEO BRCA data (GSE69914) and kept the same CpG sites for comparison purposes. We ended up with 344 947 CpGs, covering 19 271 genes, from 18 normal tissues. We then compared these normal samples to the same 42 normal-adjacent tissues from the GEO BRCA data in a replication analysis.

At the same 0.0005 threshold for gene-level *P*-values, 7 out of the 21 previously identified genes with epigenetic field defects in the discovery analysis using the GEO BRCA data were replicated by ${{{\bf D}}^{w - DM - DV}}$. The seven genes are *DPH3B, NAA35, ANKRD13B, CXCL6, FKBP4, PRSS48* and *CFTR*. We similarly validated these 7 genes by comparing *P*-values from the two-sample $t$-tests comparing the 18 replication normal samples to the 42 GEO BRCA normal-adjacent samples and *P*-values from the two-sample $t$-tests comparing the 42 GEO BRCA normal-adjacent samples to the 263 independent GEO BRCA tumor samples (Supplementary Figure S11). All 7 genes, except the *NAA35* and *FKBP4*, exhibit progression to tumor. More details of the replication analysis results using ${{{\bf D}}^{w - DM - DV}}$, $EWA{S^{\min - P}}$ and other comparing distance-based methods were summarized in Supplementary File section 3.3 Replication Analysis and Supplementary Table S8 and Supplementary Figures S12–S14.

To investigate our hypothesis that different epigenetic field defects maybe identified when comparing normal samples to a different set of normal-adjacent samples, we obtained a new set of BRCA normal-adjacent samples (n = 90) from the Cancer Genome Atlas (TCGA) project together with their matched tumor samples (*n* = 90). We plotted DNA methylation measures of CpGs in the 7 replicated genes (Supplementary File 2) of the 18 replication normal samples, the 50 discovery GEO BRCA normal samples, the 42 discovery GEO BRCA normal-adjacent samples, the 42 discovery GEO BRCA matched tumor samples, and the 90 TCGA normal-adjacent samples and the 90 TCGA matched tumor samples. It is clear that methylation patterns of the TCGA normal-adjacent tissues are very different from that of the discovery GEO BRCA normal-adjacent tissues in most of these CpGs. This supports our hypothesis that methylation patterns can be very different in different pre-cancer tissues (using normal-adjacent tissue as a surrogate) thus different epigenetic field defects maybe identified when normal samples are compared to different sets of pre-cancer tissues.

## DISCUSSION

In this study, we developed a weighted epigenetic distance-based method ${{{\bf D}}^{w - DM - DV}}$ that accumulates both DM (mean) and DV (variance) signals across CpGs in a gene or a genetic region. One known advantage of distance-based methods is, there is no need to preselect outcome-associated features, avoiding the potential to mis-screen features with weak signals. In our proposed weighted epigenetic distance-based method ${{{\bf D}}^{w - DM - DV}}$, we used CpG site-level association strengths as weights for individual CpGs aiming to up-weight signal CpGs and down-weight noise CpGs. If the feature preselection step could be conducted perfectly, it is equivalent to the case when weight ‘0’ is correctly assigned to noise CpGs and weight ‘1’ is correctly assigned to signal CpGs. Results from simulation studies suggest that when the signal-to-noise ratio in a gene decreases, power of non-weighted epigenetic distance-based methods decreased drastically, while power of the weighted version was well maintained. This suggests that incorporating CpG-site-level association strengths as weights for individual CpGs indeed help to up-weight signal CpGs and down-weight noise CpGs, thus improve the overall study performance. Simulation results also suggest that the weighted epigenetic distance-based methods will be most effective when applied to genes or genetic regions with a small percentage of CpGs having weak signals. This makes the detection of epigenetic field defects, i.e., early epigenetic alterations that are usually infrequent across samples and identifiable as outlier samples, the ideal application of the proposed method ${{{\bf D}}^{w - DM - DV}}$. Using the GEO BRCA 450K DNA methylation data, ${{{\bf D}}^{w - DM - DV}}$ identified 21 genes with epigenetic field defects, when 7 out of the 21 genes overlap with the genes identified by $EWA{S^{\min - P}}$. Majority of the genes uniquely identified by ${{{\bf D}}^{w - DM - DV}}$ were previously reported to be associated with breast cancer. Most of the genes uniquely identified by $EWA{S^{\min - P}}$ also ranked on top in the ${{{\bf D}}^{w - DM - DV}}$ results except for the *PLS1* gene. However, further investigations suggested that the *PLS1* gene may not be a real epigenetic field defect. On the other hand, most of the genes uniquely identified by ${{{\bf D}}^{w - DM - DV}}$ also ranked on top in the $EWA{S^{\min - P}}$ results except for the *CFTR* gene, in which the enrichment in the progression to breast cancer was confirmed in further analyses. This suggests that genes identified by ${{{\bf D}}^{w - DM - DV}}$, which are generally characterized by multiple signal CpGs, are more reliable. It is worth noticing that the 2 DMR-covered genes identified in our previous work (21) also ranked on top in the ${{{\bf D}}^{w - DM - DV}}$ results. We validated the identified epigenetic field defects by showing a progression to tumor in an independent dataset of tumor tissues. We also conducted a replication analysis by comparing the same set of normal-adjacent tissues to an independent set of normal tissues, and found that 7 out of the 21 genes of epigenetic field defects identified by ${{{\bf D}}^{w - DM - DV}}$ in the discovery analysis were replicated.

In general, distance-based methods have a better performance than that of site-level EWAS methods when site-level signals are weak. As discussed in our previous work (21) and work of others (3,4), epigenetic field defects are often characterized by increased variation in DNA methylation measures due to a few outlier normal-adjacent tissue samples. So the site-level EWAS methods are usually underpowered due to small mean differences as well as stringent multiple comparisons adjustment. Distance-based methods accumulate weak signals to improve power. Distance-based methods are flexible and can be applied to a CpG site, a gene, a pathway, or an entire genome. A closer investigation on what we identified in our previous work (21) in DMR detection and the current work suggests that we may take advantages of the techniques in DMR detection and combine that with distance-based methods in future works to more efficiently identify regions of epigenetic field defects.

In summary, we proposed a new weighted distance-based method ${{{\bf D}}^{w - DM - DV}}$ that considers both DM and DV in DNA methylation and incorporates site-level association strengths as weights on individual CpGs to up-weight signal CpGs and down-weight noise CpGs to further boost the overall study power. The ${{{\bf D}}^{w - DM - DV}}$ method is especially powerful in detecting epigenetic field defects when methylation alterations between normal tissues and normal-adjacent tissues are usually minimum.

## DATA AVAILABILITY

An R code for the proposed method ${{{\bf D}}^{w - DM - DV}}$ together with a tutorial and a sample data set is available for downloading from http://www.columbia.edu/∼sw2206/softwares.htm.

The BRCA 450K DNA methylation data of 50 normal tissues, 42 normal tissues adjacent to tumors together with 42 matched tumor tissues, and 263 independent tumor tissues were downloaded from Gene Expression Omnibus (GEO) under the accession number GSE69914. The 450K DNA methylation data of 18 normal tissue of 18 breast reduction mammoplasty subjects were downloaded from Gene Expression Omnibus (GEO) under the accession number GSE67919. The 450K DNA methylation data of 90 BRCA normal-adjacent and tumor pairs were downloaded from the Cancer Genome Atlas (TCGA) project.

## Supplementary Material

Supplementary Data

## References

[1] Katsurano M., Niwa T., Yasui Y., Shigematsu Y., Yamashita S., Takeshima H., Lee M., Kim Y., Tanaka T., Ushijima T. Early-stage formation of an epigenetic field defect in a mouse colitis model, and non-essential roles of T-and B-cells in DNA methylation induction. Oncogene. 2012; 31:342. 21685942 10.1038/onc.2011.241

[2] Bernstein C., Nfonsam V., Prasad A.R., Bernstein H. Epigenetic field defects in progression to cancer. World J. Gastrointestinal Oncol. 2013; 5:43. 10.4251/wjgo.v5.i3.43 PMC3648662 23671730

[3] Teschendorff A.E., Gao Y., Jones A., Ruebner M., Beckmann M.W., Wachter D.L., Fasching P.A., Widschwendter M. DNA methylation outliers in normal breast tissue identify field defects that are enriched in cancer. Nat. Commun. 2016; 7:10478. 26823093 10.1038/ncomms10478 PMC4740178

[4] Teschendorff A.E., Jones A., Widschwendter M. Stochastic epigenetic outliers can define field defects in cancer. BMC Bioinformatics. 2016; 17:1. 27103033 10.1186/s12859-016-1056-z PMC4840974

[5] Baylin S.B., Esteller M., Rountree M.R., Bachman K.E., Schuebel K., Herman J.G. Aberrant patterns of DNA methylation, chromatin formation and gene expression in cancer. Hum. Mol. Genet. 2001; 10:687–692. 11257100 10.1093/hmg/10.7.687

[6] Fahrner J.A., Eguchi S., Herman J.G., Baylin S.B. Dependence of histone modifications and gene expression on DNA hypermethylation in cancer. Cancer Res. 2002; 62:7213–7218. 12499261

[7] Jones P.A. Functions of DNA methylation: islands, start sites, gene bodies and beyond. Nat. Rev. Genet. 2012; 13:484–492. 22641018 10.1038/nrg3230

[8] Phillips T. The role of methylation in gene expression. Nat. Educ. 2008; 1:116.

[9] Das P.M., Singal R. DNA methylation and cancer. J. Clin. Oncol. 2004; 22:4632–4642. 15542813 10.1200/JCO.2004.07.151

[10] Ehrlich M. DNA methylation in cancer: too much, but also too little. Oncogene. 2002; 21:5400–5413. 12154403 10.1038/sj.onc.1205651

[11] Esteller M., Herman J.G. Cancer as an epigenetic disease: DNA methylation and chromatin alterations in human tumours. J. Pathol. 2002; 196:1–7. 11748635 10.1002/path.1024

[12] Kulis M., Esteller M. DNA methylation and cancer. Adv Genet. 2010; 70:27–56. 20920744 10.1016/B978-0-12-380866-0.60002-2

[13] Koukoura O., Spandidos D.A., Daponte A., Sifakis S. DNA methylation profiles in ovarian cancer: implication in diagnosis and therapy. Mol. Med. Rep. 2014; 10:3–9. 24821107 10.3892/mmr.2014.2221 PMC4068729

[14] Baylin S.B. DNA methylation and gene silencing in cancer. Nat. Clin. Pract. Oncol. 2005; 2:S4–S11. 16341240 10.1038/ncponc0354

[15] Curradi M., Izzo A., Badaracco G., Landsberger N. Molecular mechanisms of gene silencing mediated by DNA methylation. Mol. Cell. Biol. 2002; 22:3157–3173. 11940673 10.1128/MCB.22.9.3157-3173.2002 PMC133775

[16] Herman J.G., Baylin S.B. Gene silencing in cancer in association with promoter hypermethylation. N. Engl. J. Med. 2003; 349:2042–2054. 14627790 10.1056/NEJMra023075

[17] Robertson K.D. DNA methylation and human disease. Nat. Rev. Genet. 2005; 6:597–610. 16136652 10.1038/nrg1655

[18] Eden A., Gaudet F., Waghmare A., Jaenisch R. Chromosomal instability and tumors promoted by DNA hypomethylation. Science. 2003; 300:455–455. 12702868 10.1126/science.1083557

[19] Feinberg A.P., Tycko B. The history of cancer epigenetics. Nat. Rev. Cancer. 2004; 4:143–153. 14732866 10.1038/nrc1279

[20] Jaenisch R., Bird A. Epigenetic regulation of gene expression: how the genome integrates intrinsic and environmental signals. Nat. Genet. 2003; 33:245–254. 12610534 10.1038/ng1089

[21] Wang Y., Teschendorff A.E., Widschwendter M., Wang S. Accounting for differential variability in detecting differentially methylated regions. Brief. Bioinform. 2017; doi:10.1093/bib/bbx097. 10.1093/bib/bbx097 29912290

[22] Tusher V.G., Tibshirani R., Chu G. Significance analysis of microarrays applied to the ionizing radiation response. Proc. Natl. Acad. Sci. U.S.A. 2001; 98:5116–5121. 11309499 10.1073/pnas.091062498 PMC33173

[23] Smyth G.K. Linear models and empirical bayes methods for assessing differential expression in microarray experiments. Stat. Appl. Genet. Mol. Biol. 2004; 3:1–25. 10.2202/1544-6115.1027 16646809

[24] Wettenhall J.M., Smyth G.K. limmaGUI: a graphical user interface for linear modeling of microarray data. Bioinformatics. 2004; 20:3705–3706. 15297296 10.1093/bioinformatics/bth449

[25] Wilcoxon F. Individual comparisons by ranking methods. Biometrics Bull. 1945; 1:80–83.

[26] Hansen K.D., Timp W., Bravo H.C., Sabunciyan S., Langmead B., McDonald O.G., Wen B., Wu H., Liu Y., Diep D. Increased methylation variation in epigenetic domains across cancer types. Nat. Genet. 2011; 43:768–775. 21706001 10.1038/ng.865 PMC3145050

[27] Ho J.W., Stefani M., dos Remedios C.G., Charleston M.A. Differential variability analysis of gene expression and its application to human diseases. Bioinformatics. 2008; 24:i390–i398. 18586739 10.1093/bioinformatics/btn142 PMC2718620

[28] Phipson B., Oshlack A. DiffVar: a new method for detecting differential variability with application to methylation in cancer and aging. Genome Biol. 2014; 15:1. 10.1186/s13059-014-0465-4 PMC4210618 25245051

[29] Zapala M.A., Schork N.J. Multivariate regression analysis of distance matrices for testing associations between gene expression patterns and related variables. Proc. Natl. Acad. Sci. U.S.A. 2006; 103:19430–19435. 17146048 10.1073/pnas.0609333103 PMC1748243

[30] Wessel J., Schork N.J. Generalized genomic distance–based regression methodology for multilocus association analysis. Am. J. Hum. Genet. 2006; 79:792–806. 17033957 10.1086/508346 PMC1698575

[31] McArdle B.H., Anderson M.J. Fitting multivariate models to community data: a comment on distance‐based redundancy analysis. Ecology. 2001; 82:290–297.

[32] Anderson M.J. A new method for non‐parametric multivariate analysis of variance. Austral Ecol. 2001; 26:32–46.

[33] Han F., Pan W. Powerful multi‐marker association tests: unifying genomic distance‐based regression and logistic regression. Genet. Epidemiol. 2010; 34:680–688. 20976795 10.1002/gepi.20529 PMC3345567

[34] Friedman J., Hastie T., Tibshirani R. The Elements of Statistical Learning. 2001; NY: Springer Series in Statistics.

[35] Hansen K. IlluminaHumanMethylation450kanno. ilmn12. hg19: annotation for illumina's 450k methylation arrays. 2015; R package, version 0.2.

[36] Hair B.Y., Xu Z., Kirk E.L., Harlid S., Sandhu R., Robinson W.R., Wu M.C., Olshan A.F., Conway K., Taylor J.A. Body mass index associated with genome-wide methylation in breast tissue. Breast Cancer Res. Treat. 2015; 151:453–463. 25953686 10.1007/s10549-015-3401-8 PMC4474159

[37] Krijgsman O., Roepman P., Zwart W., Carroll J.S., Tian S., de Snoo F.A., Bender R.A., Bernards R., Glas A.M. A diagnostic gene profile for molecular subtyping of breast cancer associated with treatment response. Breast Cancer Res. Treat. 2012; 133:37–47. 21814749 10.1007/s10549-011-1683-z

[38] Tada Y., Yamaguchi Y., Kinjo T., Song X., Akagi T., Takamura H., Ohta T., Yokota T., Koide H. The stem cell transcription factor ZFP57 induces IGF2 expression to promote anchorage-independent growth in cancer cells. Oncogene. 2015; 34:752–760. 24469060 10.1038/onc.2013.599

[39] Abu-Asab M., Abu-Asab N., Loffredo C., Clarke R., Amri H. Identifying early events of gene expression in breast cancer with systems biology phylogenetics. Cytogenet. Genome Res. 2013; 139:206–214. 23548567 10.1159/000348433 PMC3671766

[40] Jönsson G., Staaf J., Vallon-Christersson J., Ringnér M., Holm K., Hegardt C., Gunnarsson H., Fagerholm R., Strand C., Agnarsson B.A. Genomic subtypes of breast cancer identified by array-comparative genomic hybridization display distinct molecular and clinical characteristics. Breast Cancer Res. 2010; 12:R42. 20576095 10.1186/bcr2596 PMC2917037

[41] Legendre C., Gooden G.C., Johnson K., Martinez R.A., Liang W.S., Salhia B. Whole-genome bisulfite sequencing of cell-free DNA identifies signature associated with metastatic breast cancer. Clinical Epigenet. 2015; 7:100. 10.1186/s13148-015-0135-8 PMC4573288 26380585

[42] Lehmann B.D., Bauer J.A., Chen X., Sanders M.E., Chakravarthy A.B., Shyr Y., Pietenpol J.A. Identification of human triple-negative breast cancer subtypes and preclinical models for selection of targeted therapies. J. Clin. Invest. 2011; 121:2750. 21633166 10.1172/JCI45014 PMC3127435

[43] Bièche I., Chavey C., Andrieu C., Busson M., Vacher S., Le Corre L., Guinebretière J.-M., Burlinchon S., Lidereau R., Lazennec G. CXC chemokines located in the 4q21 region are up-regulated in breast cancer. Endocr. Relat. Cancer. 2007; 14:1039–1052. 18045955 10.1677/erc.1.01301

[44] Hou J., Wu J., Dombkowski A., Zhang K., Holowatyj A., Boerner J.L., Yang Z.-Q. Genomic amplification and a role in drug-resistance for the KDM5A histone demethylase in breast cancer. Am. J. Transl. Res. 2012; 4:247. 22937203 PMC3426386

[45] Wolf I., Bose S., Desmond J.C., Lin B.T., Williamson E.A., Karlan B.Y., Koeffler H.P. Unmasking of epigenetically silenced genes reveals DNA promoter methylation and reduced expression of PTCH in breast cancer. Breast Cancer Res. Treat. 2007; 105:139–155. 17295047 10.1007/s10549-006-9440-4

[46] Steinbach D., Schramm A., Eggert A., Onda M., Dawczynski K., Rump A., Pastan I., Wittig S., Pfaffendorf N., Voigt A. Identification of a set of seven genes for the monitoring of minimal residual disease in pediatric acute myeloid leukemia. Clin. Cancer Res. 2006; 12:2434–2441. 16638849 10.1158/1078-0432.CCR-05-2552

[47] Cao X.-C., Zhang W.-R., Cao W.-F., Liu B.-W., Zhang F., Zhao H.-M., Meng R., Zhang L., Niu R.-F., Hao X.-S. Aquaporin3 is required for FGF-2-induced migration of human breast cancers. PLoS One. 2013; 8:e56735. 23468877 10.1371/journal.pone.0056735 PMC3585269

[48] Nicolau M., Levine A.J., Carlsson G. Topology based data analysis identifies a subgroup of breast cancers with a unique mutational profile and excellent survival. Proc. Natl. Acad. Sci. U.S.A. 2011; 108:7265–7270. 21482760 10.1073/pnas.1102826108 PMC3084136

[49] Xia T.-S., Wang G.-Z., Ding Q., Liu X.-A., Zhou W.-B., Zhang Y.-F., Zha X.-M., Du Q., Ni X.-J., Wang J. Bone metastasis in a novel breast cancer mouse model containing human breast and human bone. Breast Cancer Res. Treat. 2012; 132:471–486. 21638054 10.1007/s10549-011-1496-0

[50] Yang W.S., Moon H.-G., Kim H.S., Choi E.-J., Yu M.-H., Noh D.-Y., Lee C. Proteomic approach reveals FKBP4 and S100A9 as potential prediction markers of therapeutic response to neoadjuvant chemotherapy in patients with breast cancer. J. Proteome Res. 2011; 11:1078–1088. 22074005 10.1021/pr2008187

[51] Deng Q., Huang S. PRDM5 is silenced in human cancers and has growth suppressive activities. Oncogene. 2004; 23:4903. 15077163 10.1038/sj.onc.1207615

[52] Giussani M., Merlino G., Cappelletti V., Tagliabue E., Daidone M.G. Seminars in Cancer Biology. 2015; 35:Elsevier 3–10. 26416466 10.1016/j.semcancer.2015.09.012

[53] Matise L.A., Palmer T.D., Ashby W.J., Nashabi A., Chytil A., Aakre M., Pickup M.W., Gorska A.E., Zijlstra A., Moses H.L. Lack of transforming growth factor-β signaling promotes collective cancer cell invasion through tumor-stromal crosstalk. Breast Cancer Res. 2012; 14:R98. 22748014 10.1186/bcr3217 PMC3680921

[54] Zhang J.T., Jiang X.H., Xie C., Cheng H., Da Dong J., Wang Y., Fok K.L., Zhang X.H., Sun T.T., Tsang L.L. Downregulation of CFTR promotes epithelial-to-mesenchymal transition and is associated with poor prognosis of breast cancer. Biochim. Biophys. Acta (BBA)-Mol. Cell Res. 2013; 1833:2961–2969. 10.1016/j.bbamcr.2013.07.021 23916755

[55] Stirzaker C., Zotenko E., Song J.Z., Qu W., Nair S.S., Locke W.J., Stone A., Armstong N.J., Robinson M.D., Dobrovic A. Methylome sequencing in triple-negative breast cancer reveals distinct methylation clusters with prognostic value. Nat. Commun. 2015; 6:5899. 25641231 10.1038/ncomms6899

[56] Rudenko V., Kazakova S., Tanas A., Popa A., Nemirovchenko V., Kuznetsova E., Zaletaev D., Strelnikov V. Identification of aberrant DNA methylation in pediatric acute myeloid leukaemia by multiplex methylation sensitive PCR. Ann. Oncol. 2016; 27:doi:10.1093/annonc/mdw375.34.

[57] Wei H., Wang H., Ji Q., Sun J., Tao L., Zhou X. NRBP1 is downregulated in breast cancer and NRBP1 overexpression inhibits cancer cell proliferation through Wnt/β-catenin signaling pathway. OncoTargets Ther. 2015; 8:3721. 10.2147/OTT.S89779 PMC4685933 26715855

[58] Wolf J., Müller-Decker K., Flechtenmacher C., Zhang F., Shahmoradgoli M., Mills G., Hoheisel J., Boettcher M. An in vivo RNAi screen identifies SALL1 as a tumor suppressor in human breast cancer with a role in CDH1 regulation. Oncogene. 2014; 33:4273. 24292671 10.1038/onc.2013.515 PMC6662585

[59] Bi D., Ning H., Liu S., Que X., Ding K. Gene expression patterns combined with network analysis identify hub genes associated with bladder cancer. Comput. Biol. Chem. 2015; 56:71–83. 25889321 10.1016/j.compbiolchem.2015.04.001

[60] Abildgaard M.O., Borre M., Mortensen M.M., Ulhøi B.P., Tørring N., Wild P., Kristensen H., Mansilla F., Ottosen P.D., Dyrskjøt L. Downregulation of zinc finger protein 132 in prostate cancer is associated with aberrant promoter hypermethylation and poor prognosis. Int. J. Cancer. 2012; 130:885–895. 21445975 10.1002/ijc.26097

[61] Castaneda F., Rosin-Steiner S., Jung K. Functional genomics analysis of low concentration of ethanol in human hepatocellular carcinoma (HepG2) cells. Role of genes involved in transcriptional and translational processes. Int. J. Med. Sci. 2007; 4:28. 10.7150/ijms.4.28 PMC1752234 17211498

[62] Fidalgo F., Rodrigues T.C., Pinilla M., Silva A.G., do Socorro Maciel M., Rosenberg C., de Andrade V.P., Carraro D.M., Krepischi A.C.V. Lymphovascular invasion and histologic grade are associated with specific genomic profiles in invasive carcinomas of the breast. Tumor Biol. 2015; 36:1835–1848. 10.1007/s13277-014-2786-z PMC4375298 25391423

