# Supplementary material for: Detection of epigenetic field defects using a weighted epigenetic distance-based method
Source: Nucleic Acids Res. 2018 Oct 10;47(1):e6. doi: 10.1093/nar/gky882 (PMC6326818; doi:10.1093/nar/gky882)
Supplement: Supplementary Data [file gky882_supplemental_files.zip › 20180914_revision_supplementary_file.pdf]

Supplementary Data for "Detection of epigenetic field defects using weighted epigenetic distance-based method"

Ya Wang[1], Min Qian[1], Peifeng Ruan[2], Andrew E. Teschendorff[3,4,5] and Shuang Wang[1*]

1. Department of Biostatistics, Mailman School of Public Health, Columbia University.

2. Department of Biostatistics, George Washington University.

3. Department of Womens Cancer, University College London.

4. CAS Key Lab of Computational Biology, Shanghai Institute for Biological Sciences, Chinese Academy of Sciences.

5. Statistical Cancer Genomics, UCL Cancer Institute, University College London.

*Corresponding author. Department of Biostatistics, Mailman School of Public Health, Columbia University, 722 West 168th Street, New York, NY 10032, USA. E-mail: sw2206@columbia.edu

1 Values of shape parameters in simulations

We set $a_0 = 46.36$ and $b_0 = 52.28$ for all CpGs in controls and noise CpGs in cases such that a beta distirbution Beta(a_0, b_0) has a mean 0.47 and a SD 0.05, mimicing the real GEO methylation data. The values of a_1 and b_1 for signal CpGs in cases and the mean and SD of the corresponding beta distributions are summarized in Supplementary Table S1.

Table S1. Values of a_1 and b_1 for signal CpGs

Scenario	a_1	b_1	Beta distribution mean	Beta distribution SD	Mean difference[1]	SD ratio[2]
Variance signal only	29.5	33.27	0.47	0.0625	0	1.25
	20.34	22.94	0.47	0.075	0	1.50
	14.82	16.71	0.47	0.0875	0	1.75
	11.24	12.67	0.47	0.10	0	2
	8.78	9.90	0.47	0.1125	0	2.25
	7.02	7.92	0.47	0.125	0	2.50
Mean signal only	48.49	50.47	0.49	0.05	0.02	1
	50.47	48.49	0.51	0.05	0.04	1
	52.28	46.36	0.53	0.05	0.06	1
	53.90	44.10	0.55	0.05	0.08	1
	55.31	41.73	0.57	0.05	0.10	1

[1]Mean difference in the signal CpG between cases and controls.
[2]SD ratio in the signal CpG between cases and controls.

2 Simulation settings with one gene considering correlations among CpGs

We conducted simulation studies to investigate the impact of correlations among neighboring CpGs on the performance of the proposed distance-based method. We simulated DNA methylation M-values which are the logit2 transformation of methylation β-values, and considered AR(1) correlation among CpGs in a gene with a correlation coefficient ρ=0.5. Here we only considered one gene for illustration purposes, and conducted simulation studies parallel as that for methylation β-values in the main text. More specifically, We considered one gene with different signal-to-noise ratios ranging from 1:0, 1:24, 1:49, 3:47, to 5:45. We considered scenarios when signal CpGs have different mean or variance signals by varying means and SDs of a normal distribution used to generate methylation M-values. We considered scenarios when mean differences in methylation M-values between cases and controls are $0.25 \times SD_0$, $0.5 \times SD_0$, $0.75 \times SD_0$, $1 \times SD_0$ and $1.25 \times SD_0$ where SD_0 is the SD in controls. We also considered scenarios when ratios of SDs for cases and controls are 1.25, 1.50, 1.75, 2 and 2.25.

Type I error rates are well controlled at the 0.05 significance level in all scenarios (Supplementart Table S2). Power results are summarized in Supplementary Figure S1 where we note that the power patterns are very similar to those observed in simulations based on methylation β-values without considering correlations among CpG sites. This implies that the correlations among neighboring CpGs in a gene do not have much impact on the performance of the proposed distance-based method, neither does the use of methylation M-values or β-values.

Table S2. Type I error rates in simulation settings with AR(1) correlation among neighboring CpGs with $\rho = 0.5$

Method	1 CpG	25 CpGs	50 CpGs
$\boldsymbol{D}^{w-DM-DV}$	0.044	0.044	0.042
$\boldsymbol{D}^{w-DM}$	0.057	0.050	0.040
$\boldsymbol{D}^{w-DV}$	0.042	0.053	0.042
$\boldsymbol{D}^{DM-DV}$	0.044	0.043	0.042
$\boldsymbol{D}^{DM}$	0.057	0.048	0.043
$\boldsymbol{D}^{DV}$	0.043	0.057	0.048
$EWAS^{DM}$	0.054	0.044	0.046
$EWAS^{DV}$	0.052	0.039	0.032

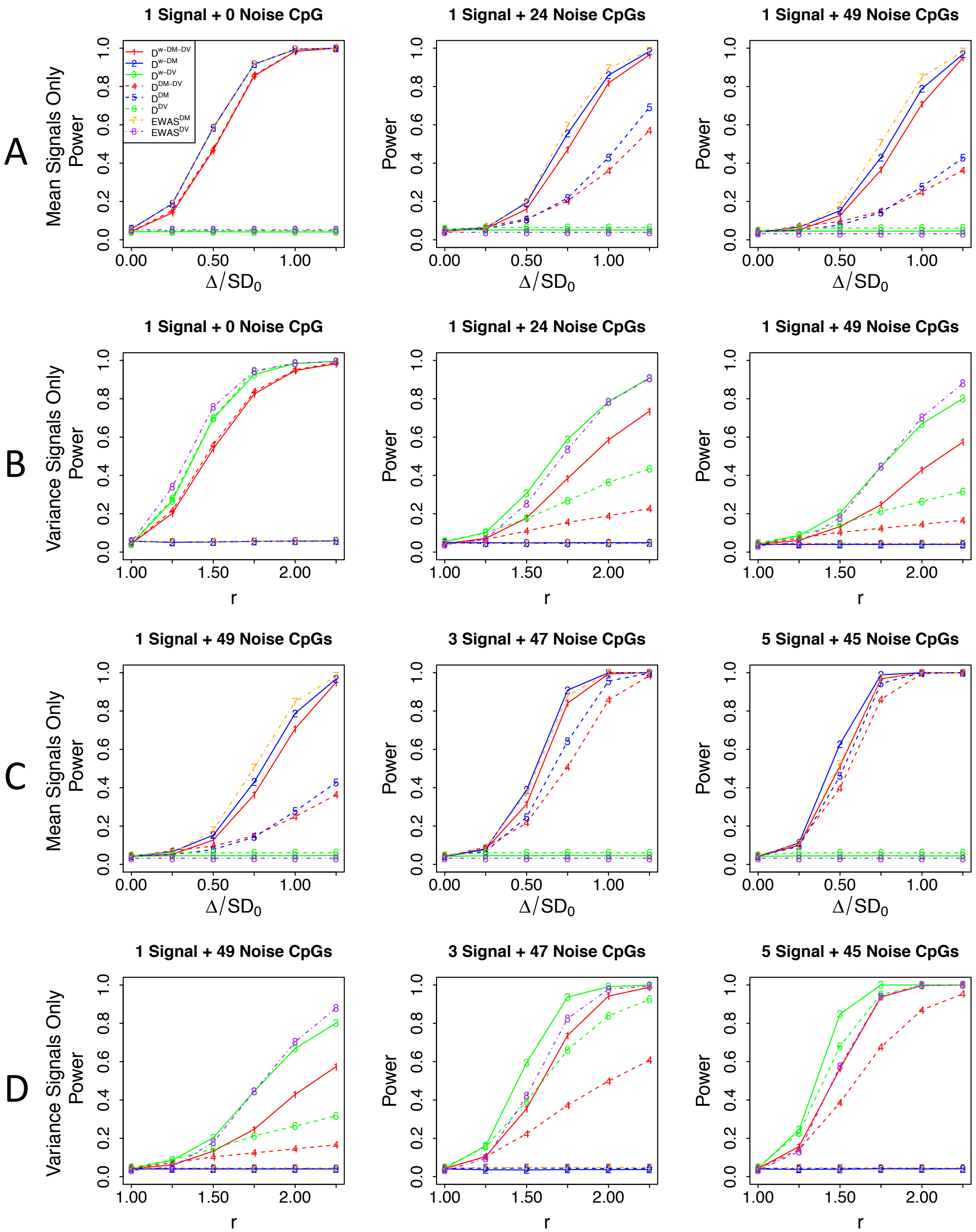

Figure S1. Power results for simulation settings with one gene considering AR(1) correlation among neighboring CpGs with correlation coefficient ρ=0.5. The signal gene has one signal CpG and increasing number of total CpGs, i.e., decreasing signal-to-noise ratios from 1:0, 1:24 to 1:49 (panel A for mean signals only, panel B for variance signals only), or with a fixed total number of CpGs 50 and increasing signal-to-noise ratios from 1:49, 3:47, to 5:45 (panel C for mean signals only, panel D for variance signals only).

3 Real data application

3.1 Discovery analysis

Table S3. 11 genes identified by $\boldsymbol{D}^{w-DM}$ at the 0.0005 gene-level p-value threshold in the GEO BRCA Data

Rank	Gene	# CpG	Rank in $\boldsymbol{D}^{w-DM-DV}$	Rank in $\boldsymbol{EWAS}^{min-P}$
1	*ZFP57**	5	2	16
2	*RGL3*	21	45	66
3	*ANKRD13B**	22	5	25
4	*PENK**	23	6	37
5	*MMP23B**	2	17	80
6	*MIR564*	9	86	55
7	*HBA1**	7	10	23
8	*SSTR4*	9	29	197
9	*PPP3R1*	11	30	299
10	*TRH**	16	13	28
11	*SOX1*	28	33	86

*Genes also identifed by $\boldsymbol{D}^{w-DM-DV}$.

Table S4. 9 genes identified by $\boldsymbol{D}^{w-DV}$ at the 0.0005 gene-level p-value threshold in the GEO BRCA Data

Rank	Gene	# CpG	Rank in $\boldsymbol{D}^{w-DM-DV}$	Rank in $\boldsymbol{EWAS}^{min-P}$
1	*KDM5A**	2	9	4
2	*CXCL6**	7	8	1
3	*DPH3B**	5	3	61
4	*TMC4**	13	1	2
5	*ANGPTL3*	3	158	50
6	*IL4R*	12	46	31
7	*NAA35**	7	4	10
8	*TMEFF1**	5	18	156
9	*THY1**	19	7	13

*Genes also identifed by $\boldsymbol{D}^{w-DM-DV}$.

Table S5. 2 significant genes identified by $\boldsymbol{D}^{DM-DV}$ at the 0.0005 gene-level p-value threshold in the GEO BRCA Data

Rank	Gene	# CpG	Rank in $\boldsymbol{D}^{w-DM-DV}$	Rank in $\boldsymbol{EWAS}^{min-P}$
1	*MMP23B**	2	17	80
2	*ZNF154*	12	26	202

*Genes also identifed by $\boldsymbol{D}^{w-DM-DV}$.

Table S6. 6 genes identified by $\boldsymbol{D}^{DM}$ at the 0.0005 gene-level p-value threshold in the GEO BRCA Data

Rank	Gene	# CpG	Rank in $\boldsymbol{D}^{w-DM-DV}$	Rank in $\boldsymbol{EWAS}^{min-P}$
1	*MMP23B**	2	17	80
2	*ZNF154*	12	26	202
3	*TRH**	16	13	28
4	*SOX17*	18	59	609
5	*WFDC3*	2	392	106
6	*SPAG6**	16	11	170

*Genes also identifed by $\boldsymbol{D}^{w-DM-DV}$.

Table S7. 4 significant genes identified by $\boldsymbol{D}^{DV}$ at the 0.0005 gene-level p-value threshold in the GEO BRCA Data

Rank	Gene	# CpG	Rank in $\boldsymbol{D}^{w-DM-DV}$	Rank in $\boldsymbol{EWAS}^{min-P}$
1	*C7orf11*	1	386	126
2	*MIR1305*	1	443	78
3	*KDM5A**	2	9	4
4	*ANGPTL3*	3	158	50

*Genes also identifed by $\boldsymbol{D}^{w-DM-DV}$.

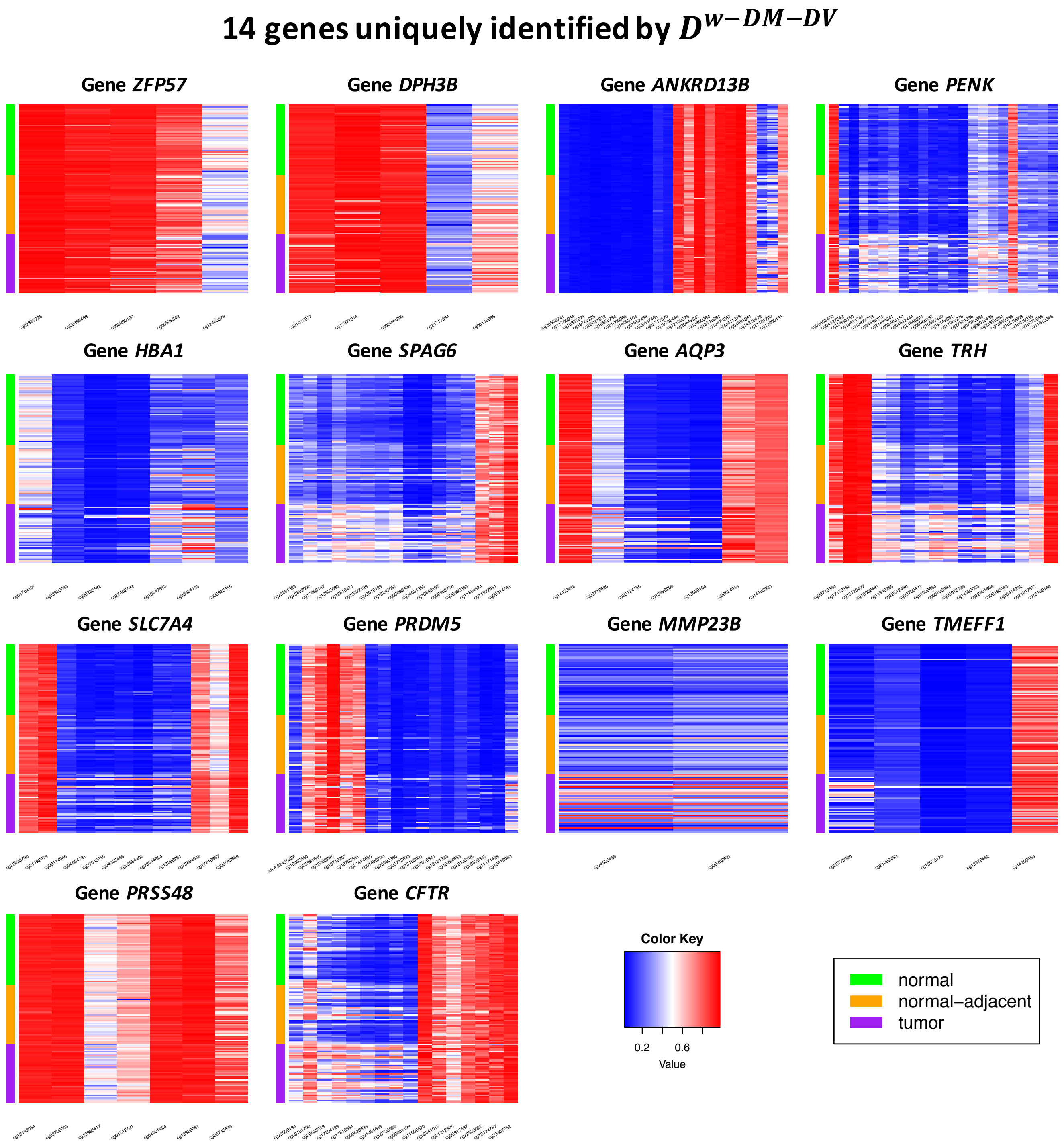

Figure S2. Heatmaps of original DNA methylation measures of the 50 normal tissues, 42 normal-adjacent tissues together with the 42 matched tumor tissues for 14 genes uniquely identified by $D^{w-DM-DV}$.

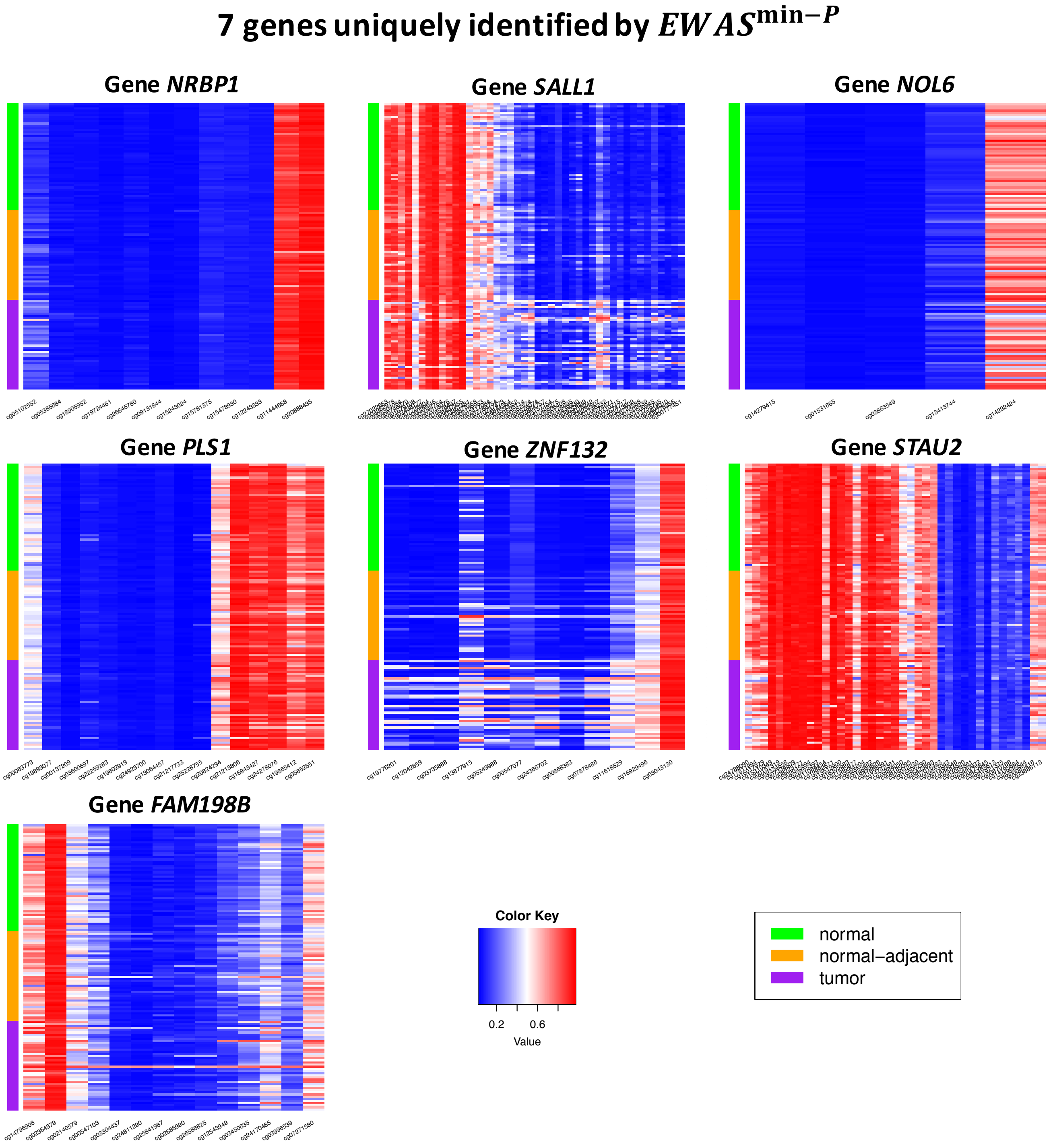

Figure S3. Heatmaps of original DNA methylation measures of the 50 normal tissues, 42 normal-adjacent tissues together with the 42 matched tumor tissues for 7 genes uniquely identified by $EWAS^{min-P}$.

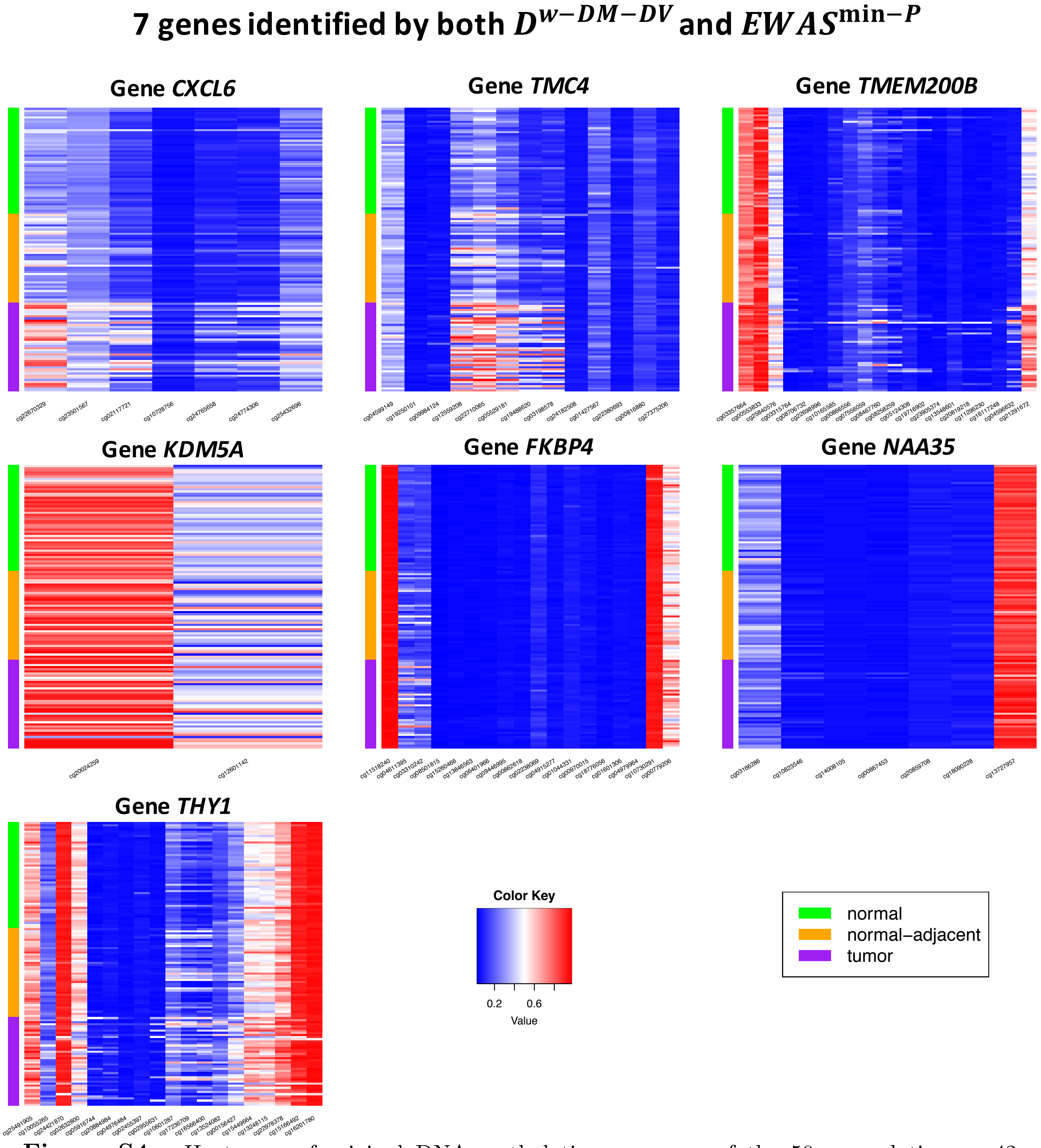

Figure S4. Heatmaps of original DNA methylation measures of the 50 normal tissues, 42 normal-adjacent tissues together with the 42 matched tumor tissues for 7 genes identified by both $\boldsymbol{D}^{w-DM-DV}$ and $EWAS^{min-P}$.

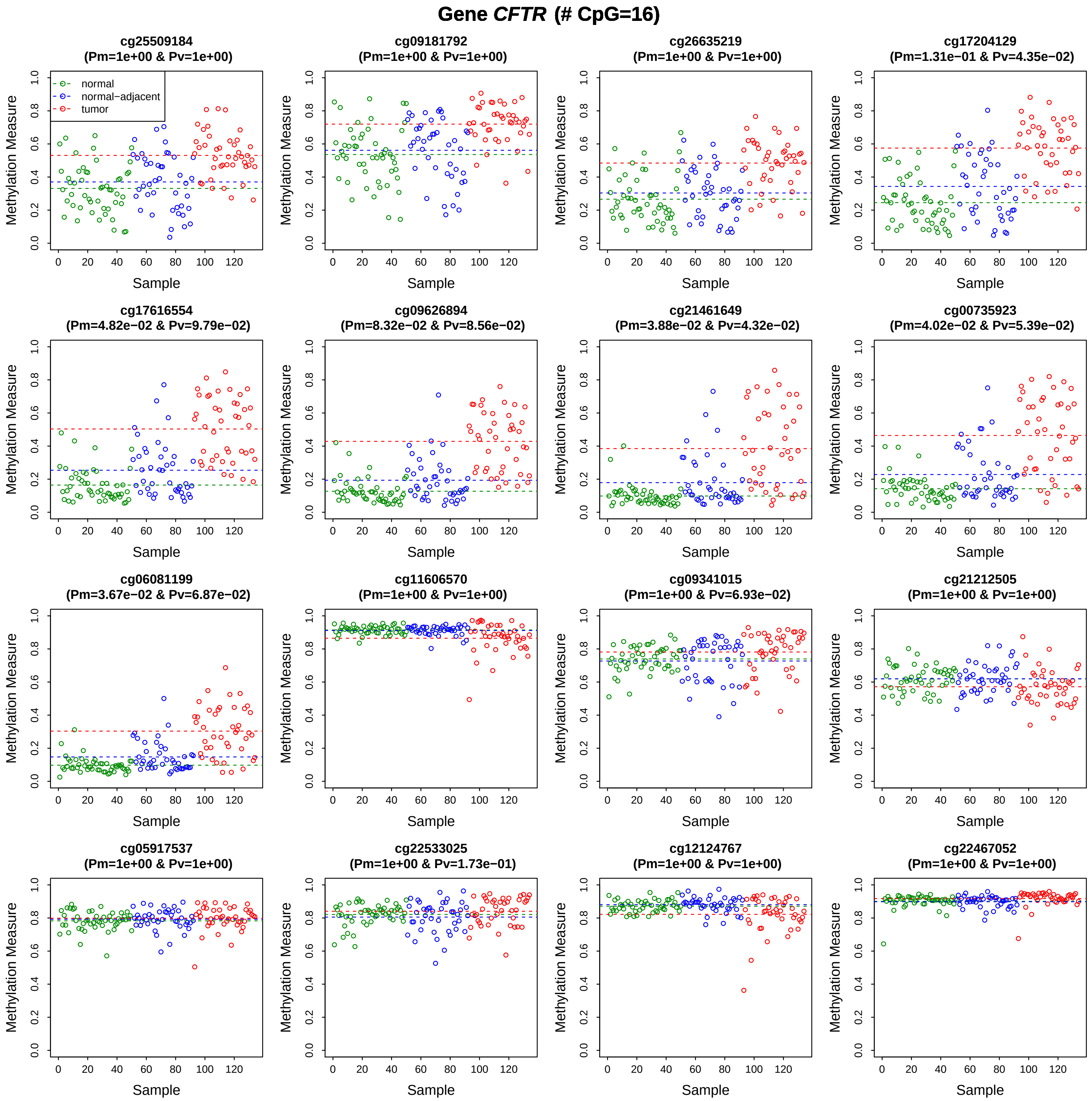

Figure S5. DNA methylation measures of 50 normal tissues, 42 normal-adjacent tissues and 42 matched tumors of 16 CpGs in the *CFTR* gene that was uniquely identified by $\boldsymbol{D}^{w-DM-DV}$, but ranked the last using $EWAS^{min-P}$ among all uniquely identified genes. Pm and Pv are p-values from CpG site-level mean and variance tests that are adjusted for multiple comparisons for the number of CpGs in the gene. The three horizontal lines represent mean methylation levels of the three groups of normal tissues, normal-adjacent tissues and matched tumors.

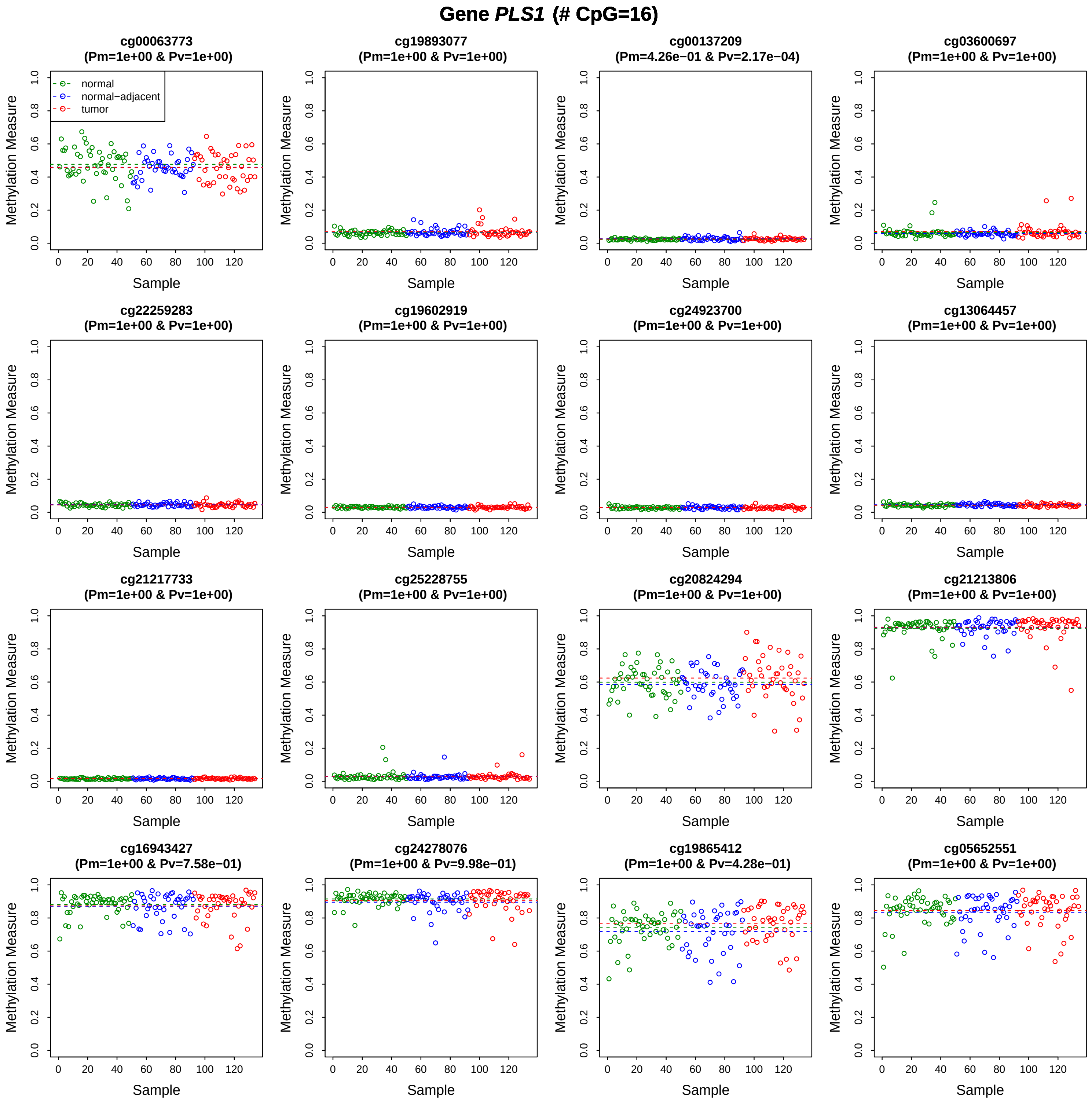

Figure S6. DNA methylation measures of 50 normal tissues, 42 normal-adjacent tissues and 42 matched tumors of 16 CpGs in the *PLS1* gene that was uniquely identified by $EWAS^{min-P}$, but ranked the last using $\boldsymbol{D}^{w-DM-DV}$ among all uniquely identified genes. Pm and Pv are *p*-values from CpG site-level mean and variance tests that are adjusted for multiple comparisons for the number of CpGs in the gene. The three horizontal lines represent mean methylation levels of the three groups of normal tissues, normal-adjacent tissues and matched tumors.

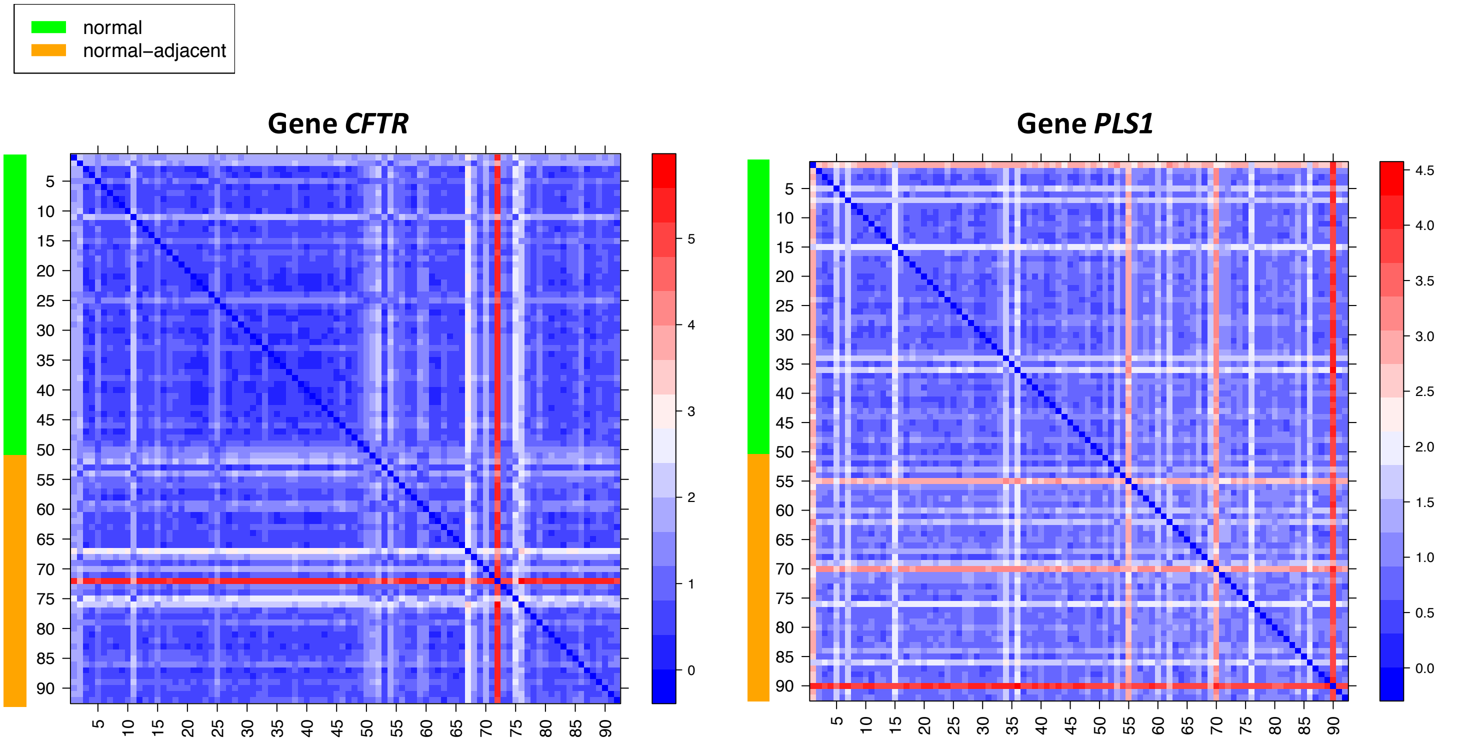

Figure S7. Weighted distance matrices for genes *CFTR* and *PLS1*.

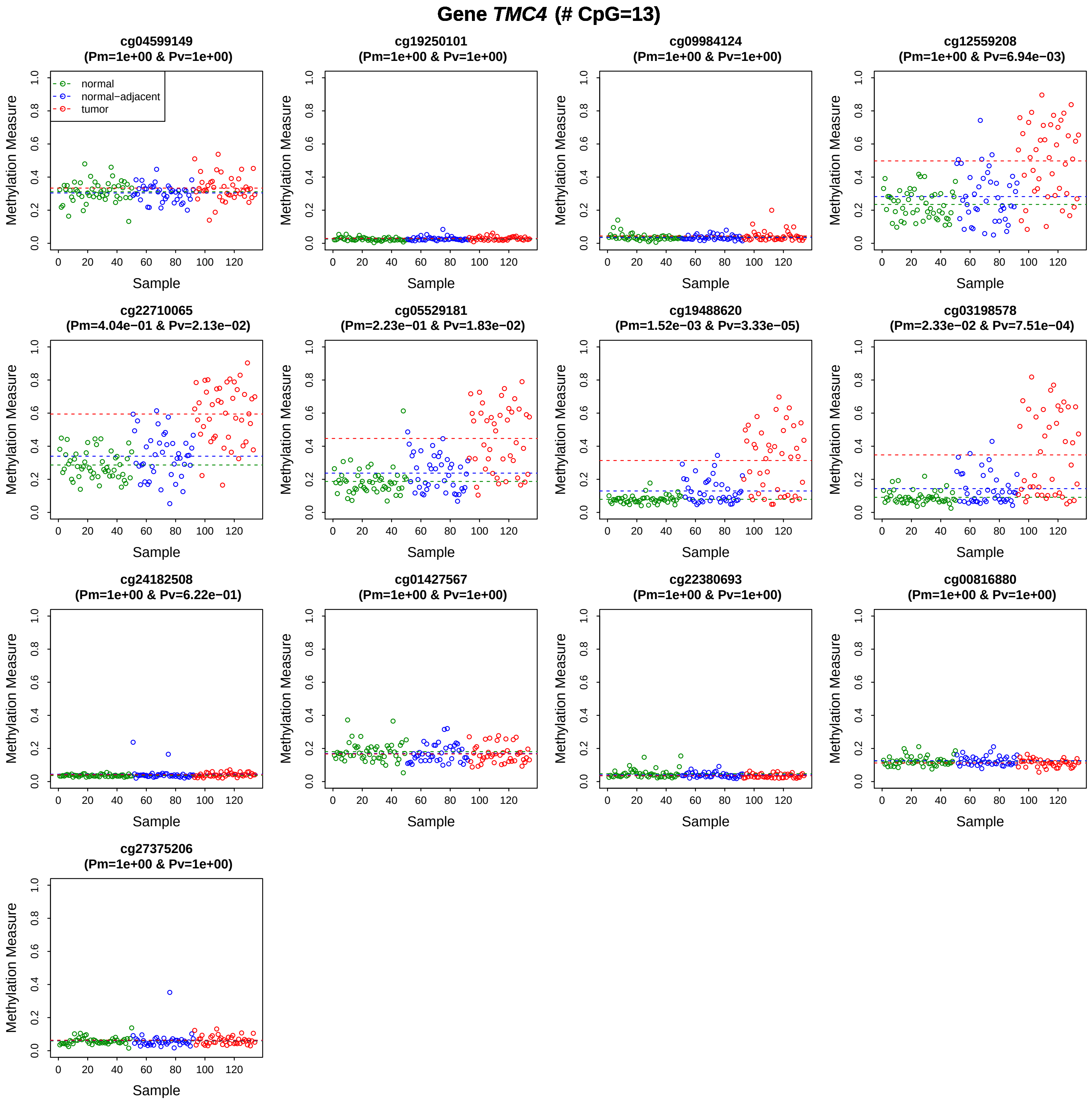

Figure S8. DNA methylation measures of 50 normal tissues, 42 normal-adjacent tissues and 42 matched tumors of 13 CpGs in the *TMC4* gene that was identified by both $\boldsymbol{D}^{w-DM-DV}$ and $EWAS^{min-P}$ and ranked on #1 and #2, respectively. Pm and Pv are *p*-values from CpG site-level mean and variance tests that are adjusted for multiple comparisons for the number of CpGs in the gene. The three horizontal lines represent mean methylation levels of the three groups of normal tissues, normal-adjacent tissues and matched tumors.

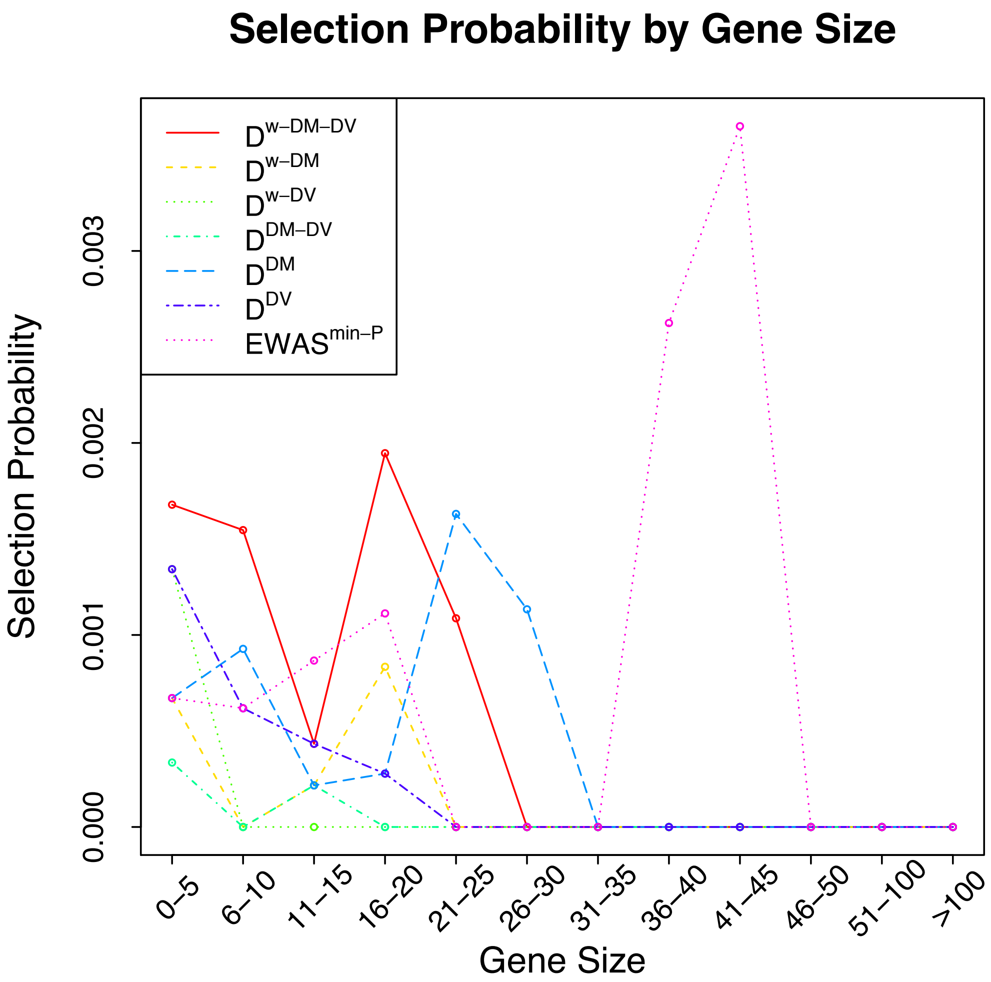

Figure S9. The selection probability of identifying a gene out of all genes of the same size.

3.2 Validation analysis

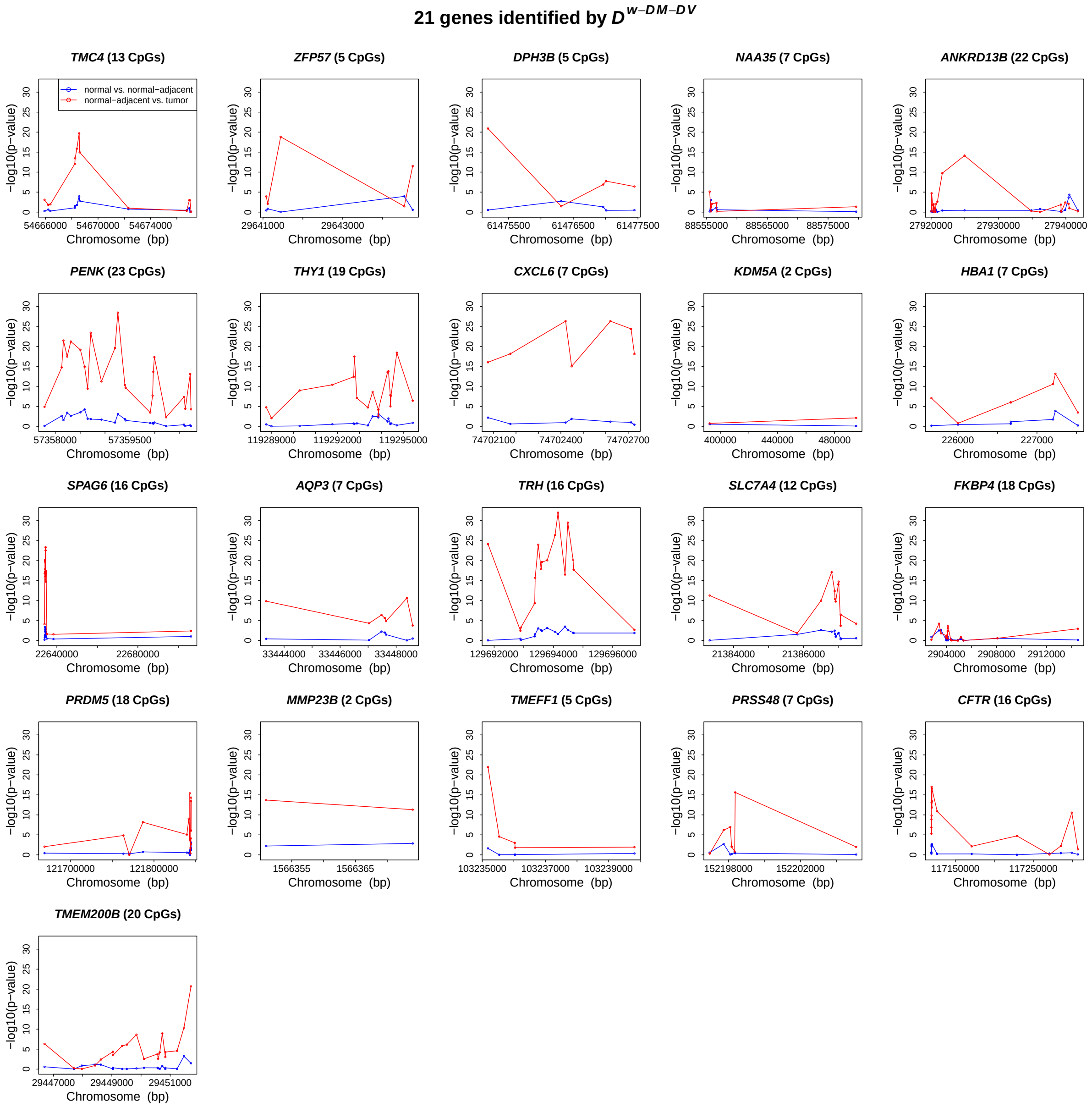

Figure S10. $-log_{10}(p\text{-value})$ from CpG site-level t-tests in (1) normal-adjacent versus normal comparison and (2) unmatched tumor versus normal-adjacent comparison in the GEO BRCA data for 21 genes identified by $\boldsymbol{D}^{w-DM-DV}$.

3.3 Replication analysis

Table S8. Summary number of genes identified by comparing methods in both the discovery analysis and replication analysis at the 0.0005 gene-level p-value threshold

Method	# of gene replicated / identified in discovery data	Replicated genes
$\boldsymbol{D}^{w-DM-DV}$	7/21	*DPH3B, NAA35, ANKRD13B, CXCL6, FKBP4, PRSS48, CFTR*
$\boldsymbol{D}^{w-DM}$	4/11	*ANKRD13B, MMP23B, PPP3R1, MIR564*
$\boldsymbol{D}^{w-DV}$	1/9	*ANGPTL3*
$\boldsymbol{D}^{DM-DV}$	2/2	*MMP23B, ZNF154*
$\boldsymbol{D}^{DM}$	2/6	*MMP23B, ZNF154*
$\boldsymbol{D}^{DV}$	1/4	*C7orf11*
$EWAS^{min-P}$	11/14	*NAA35, THY1, CXCL6, FKBP4, TMEM200B, FAM198B, NRBP1, NOL6, STAU2, SALL1, PLS1*

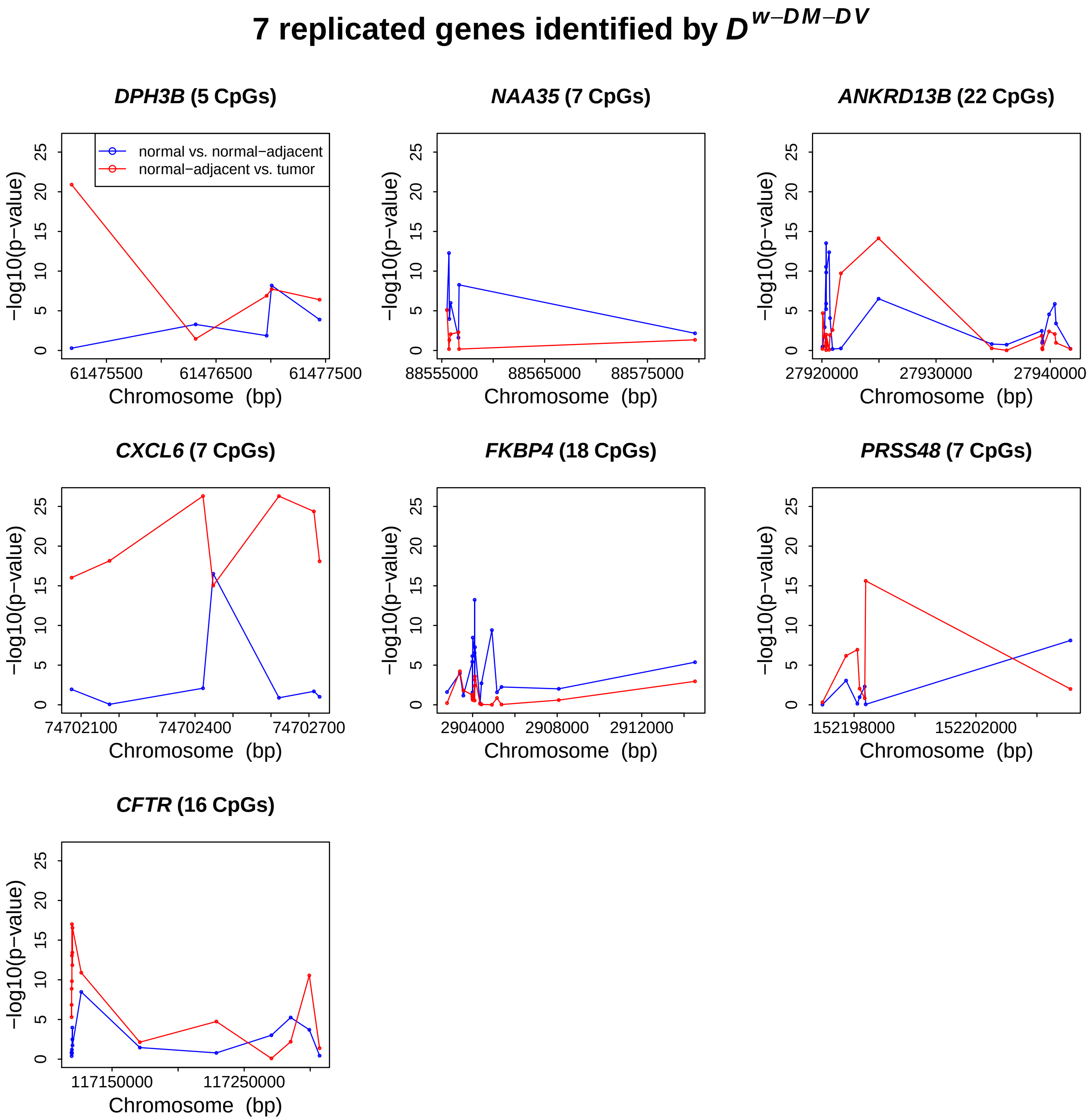

Figure S11. $-log_{10}$(p-value) from CpG site-level t-tests in (1) normal-adjacent versus normal comparison and (2) unmatched tumor versus normal-adjacent comparison in the replication analysis for 7 replicated genes identified by $\boldsymbol{D}^{w-DM-DV}$.

We plotted the DNA methylation measures for 18 normal samples from the replication data (GSE67919), 50 normal samples, 42 normal-adjacent samples and 42 matched tumor samples from the discovery data (GSE69914) (Supplementary Figure S12) for all CpGs in the *CFTR* gene. In the discovery analysis, the *CFTR* gene was uniquely identified by $\boldsymbol{D}^{w-DM-DV}$ but ranked the last using $EWAS^{min-P}$ among all $\boldsymbol{D}^{w-DM-DV}$ uniquely identified genes. The *CFTR* gene was replicated in the replication data by $\boldsymbol{D}^{w-DM-DV}$ due to weak dense signals similarly as in the discovery analysis. As the second example, we plotted the DNA methylation measures of all CpGs in the *CXCL6* gene, which was identified in the discovery analysis by both $\boldsymbol{D}^{w-DM-DV}$ and $EWAS^{min-P}$ and was replicated by both methods in the replication data (Supplementary Figure S13). It is clear that all CpGs in the *CXCL6* gene have weak signals, and some of these weak dense signals were mainly due to a few outlier normal-adjacent tissue samples which was also observed in the discovery analysis. All CpGs in the *CXCL6* gene showed enrichment in methylation measures in the progression to tumor.

Among the 14 genes identified by $EWAS^{min-P}$ in the discovery analysis, 11 were replicated in the replication data, which are *CXCL6*, *TMEM200B*, *NRBP1*, *SALL1*, *FKBP4*, *NOL6*, *PLS1*, *NAA35*, *STAU2*, *THY1*, and *FAM198B*. We similarly plotted the DNA methylation measures of all CpGs in the *PLS1* gene (Supplementary Figure S14) of the samples in the replication data. In the discovery analysis, the *PLS1* gene was uniquely identified by $EWAS^{min-P}$ but ranked the last using $\boldsymbol{D}^{w-DM-DV}$ among all $EWAS^{min-P}$ uniquely identified genes, and we found that it was identified mainly due to the strong variance signal at the CpG site cg00137209 as a result of very small variation in the methylation measures of the 50 normal tissues in the discovery data. In the replication analysis, however, the *PLS1* gene was replicated due to the mean signal at a different CpG site cg21213806. This implies that the *PLS1* gene might not be reliable, even it was replicated.

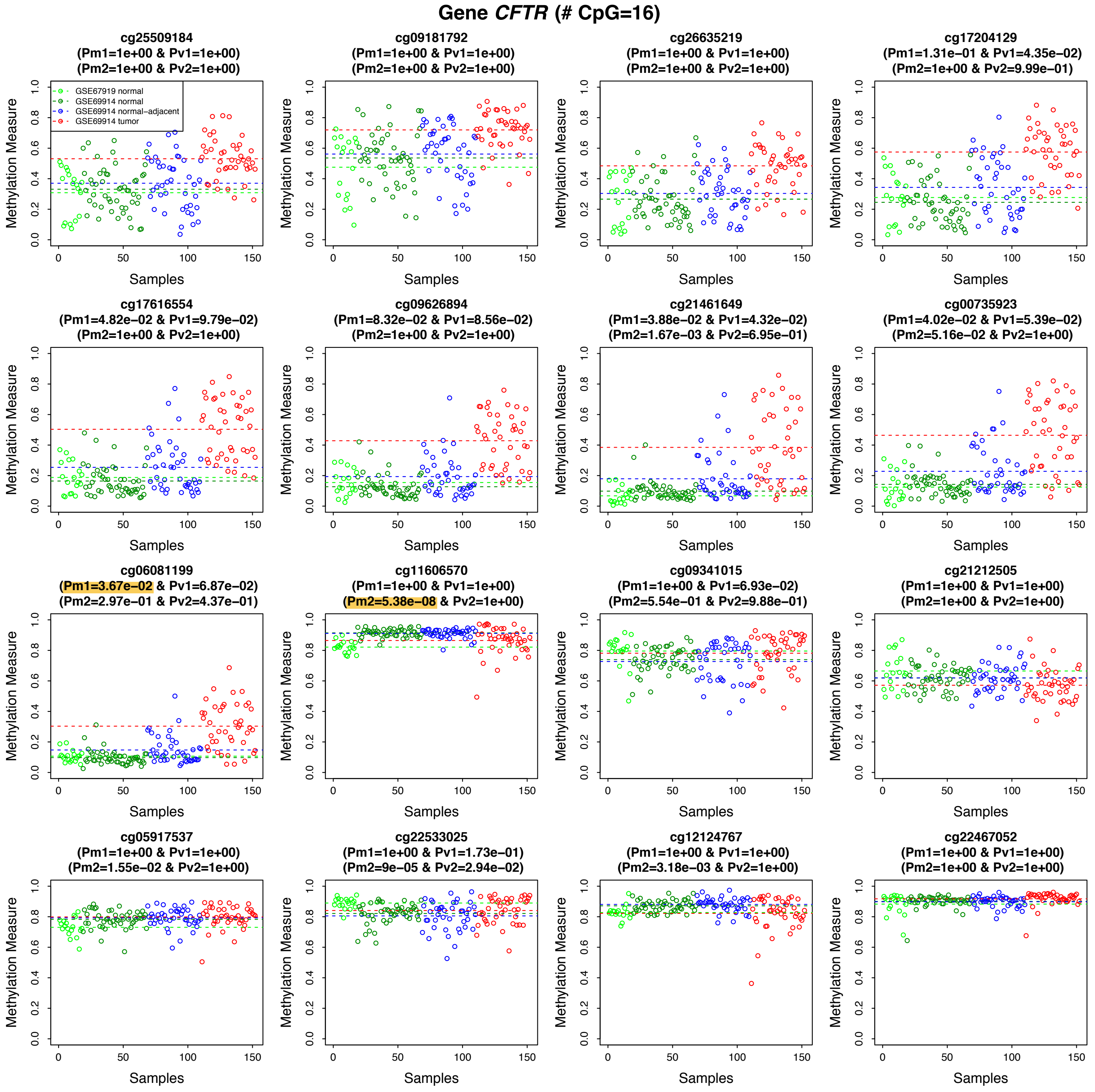

Figure S12. DNA methylation measures of 18 normal tissues from the replication data (GSE67919), 50 normal tissues, 42 normal-adjacent tissues and 42 matched tumors from the discovery data (GSE69914) of 16 CpGs in the *CFTR* gene that was uniquely identified in the discovery analysis and replicated by $\boldsymbol{D}^{w-DM-DV}$. Pm1 and Pv1 are p-values from CpG site-level mean and variance tests that are adjusted for multiple comparisons for the number of CpGs in the gene from the discovery analysis, and Pm2 and Pv2 are those from the replication analysis. Highlighted are the minimum adjusted DM and DV p-value across all p-values in the gene in each comparison. The four horizontal lines represent mean methylation levels of the four groups of tissues.

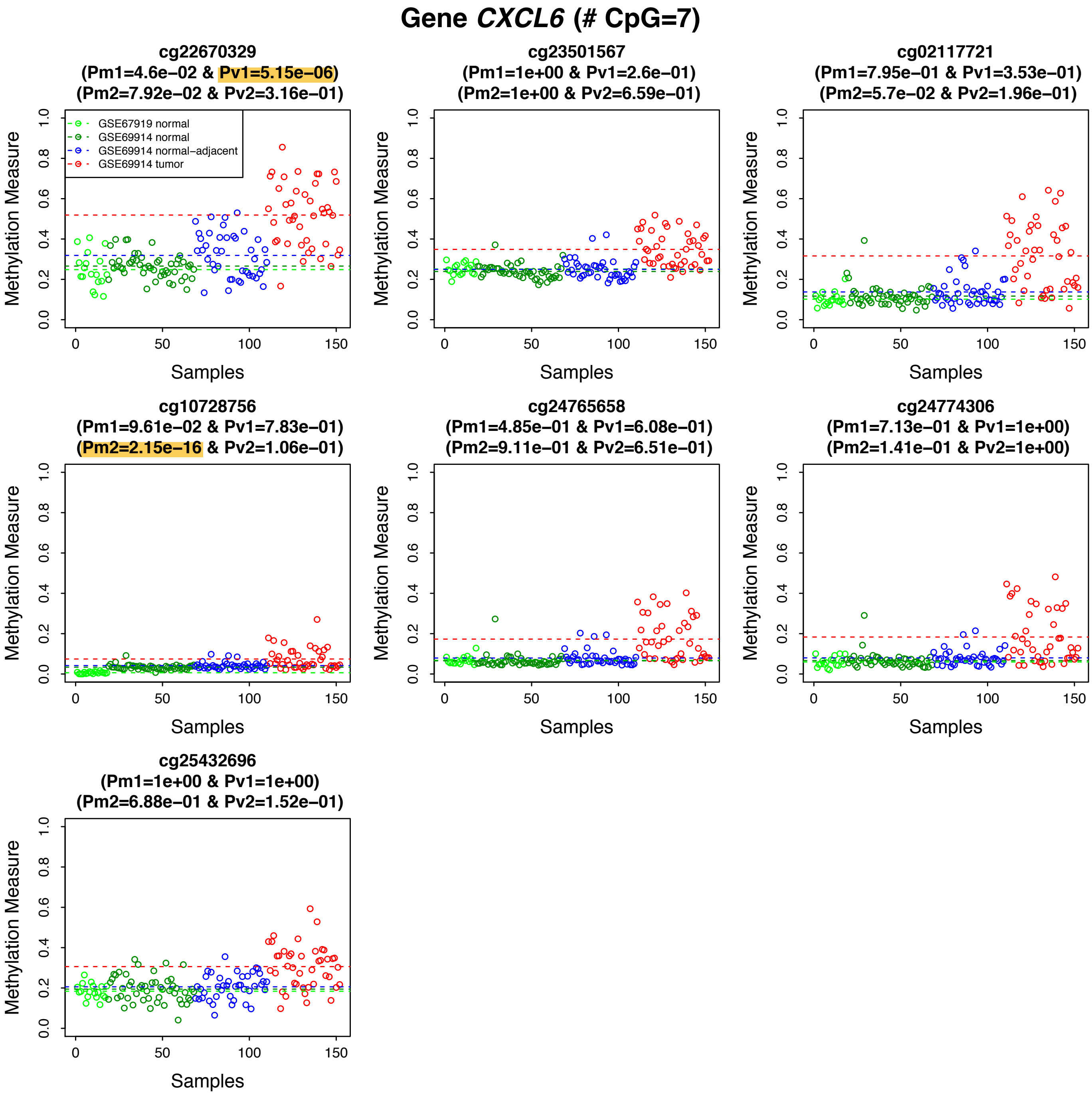

Figure S13. DNA methylation measures of 18 normal tissues from the replication data (GSE67919), 50 normal tissues, 42 normal-adjacent tissues and 42 matched tumors from the discovery data (GSE69914) of 7 CpGs in the *CXCL6* gene which was identified in the discovery analysis and replicated by both $\boldsymbol{D}^{w-DM-DV}$ and $EWAS^{min-P}$. Pm1 and Pv1 are p-values from CpG site-level mean and variance tests that are adjusted for multiple comparisons for the number of CpGs in the gene from the discovery analysis, and Pm2 and Pv2 are those from the replication analysis. Highlighted are the minimum adjusted DM and DV p-value across all p-values in the gene in each comparison. The four horizontal lines represent mean methylation levels of the four groups of tissues.

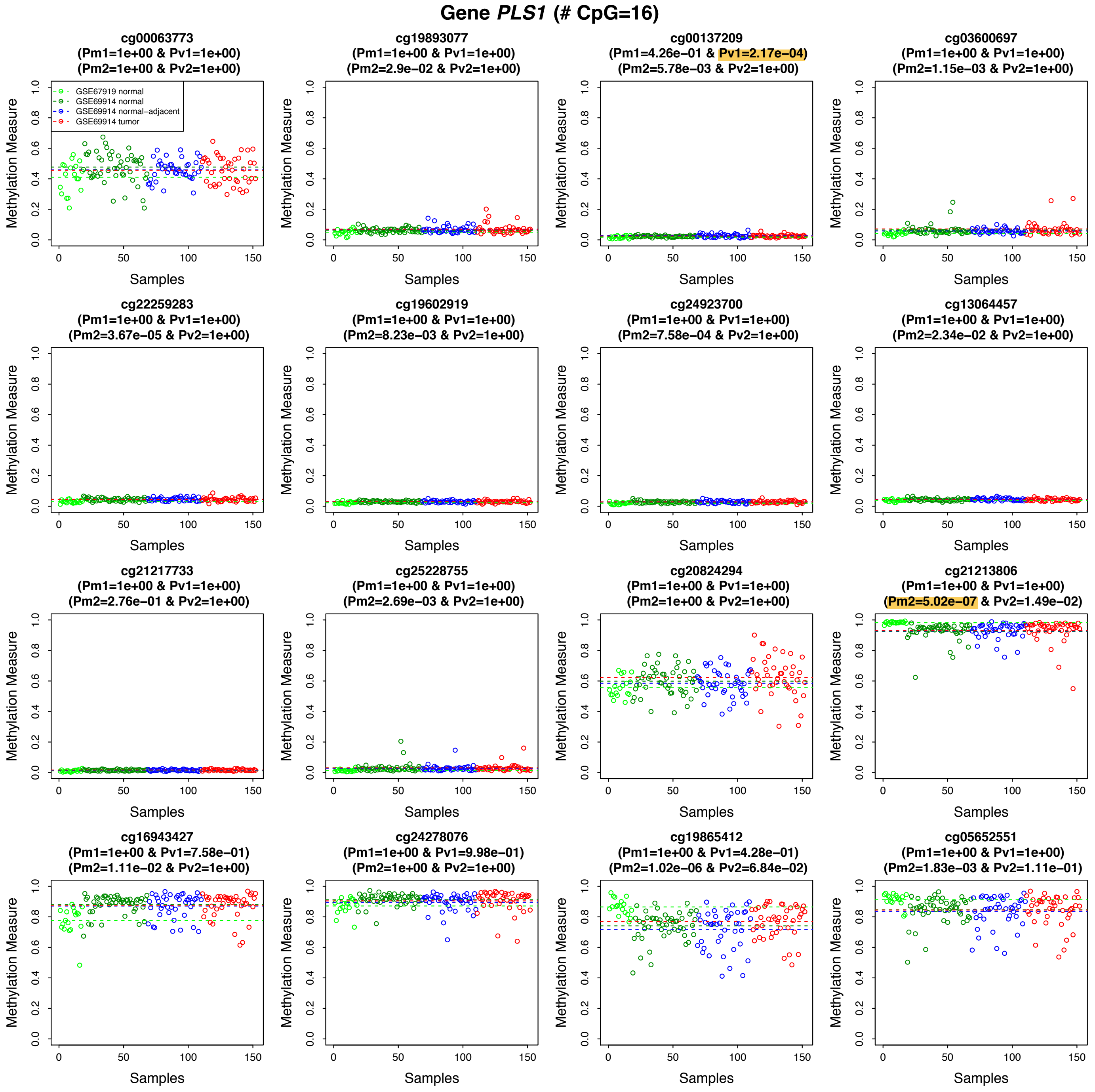

Figure S14. DNA methylation measures of 18 normal tissues from the replication data (GSE67919), 50 normal tissues, 42 normal-adjacent tissues and 42 matched tumors from the discovery data (GSE69914) of 16 CpGs in the *PLS1* gene that was uniquely identified in the discovery analysis and replicated by $EWAS^{min-P}$. Pm1 and Pv1 are p-values from CpG site-level mean and variance tests that are adjusted for multiple comparisons for the number of CpGs in the gene from the discovery analysis, and Pm2 and Pv2 are those from the replication analysis. Highlighted are the minimum adjusted DM and DV p-value across all p-values in the gene in each comparison. The four horizontal lines represent mean methylation levels of the four groups of tissues.
