# Supplementary material for: Detection of epigenetic field defects using a weighted epigenetic distance-based method
Source: Nucleic Acids Res. 2018 Oct 10;47(1):e6. doi: 10.1093/nar/gky882 (PMC6326818; doi:10.1093/nar/gky882)
Supplement: Supplementary Data [file gky882_supplemental_files.zip › Revision_supplementary_file_2.pdf]

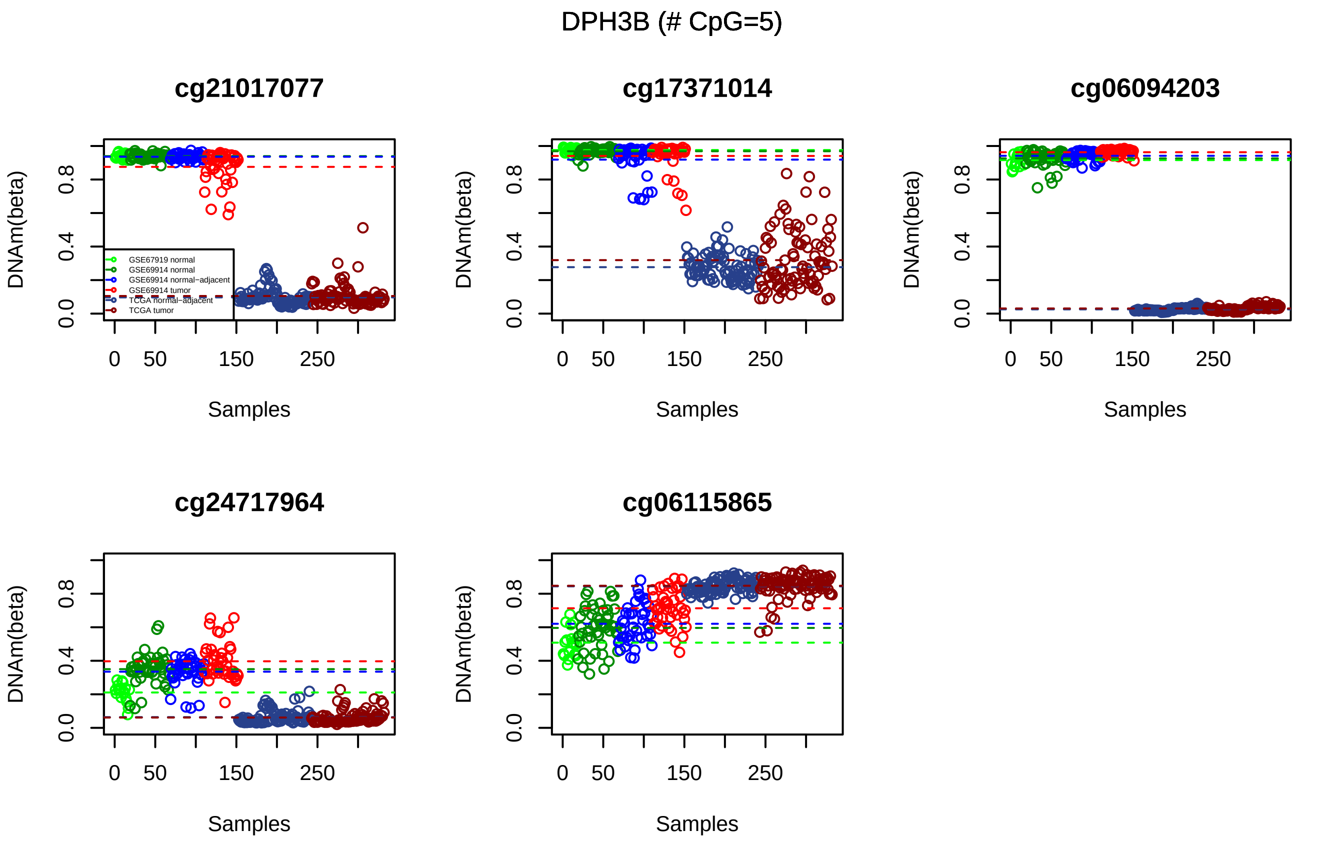

DPH3B (# CpG=5)
cg21017077
cg17371014
cg06094203
cg24717964
cg06115865
DNAm(beta)
Samples
0.0
0.4
0.8
0
50
150
250
GSE67919 normal
GSE69914 normal
GSE69914 normal−adjacent
GSE69914 tumor
TCGA normal−adjacent
TCGA tumor

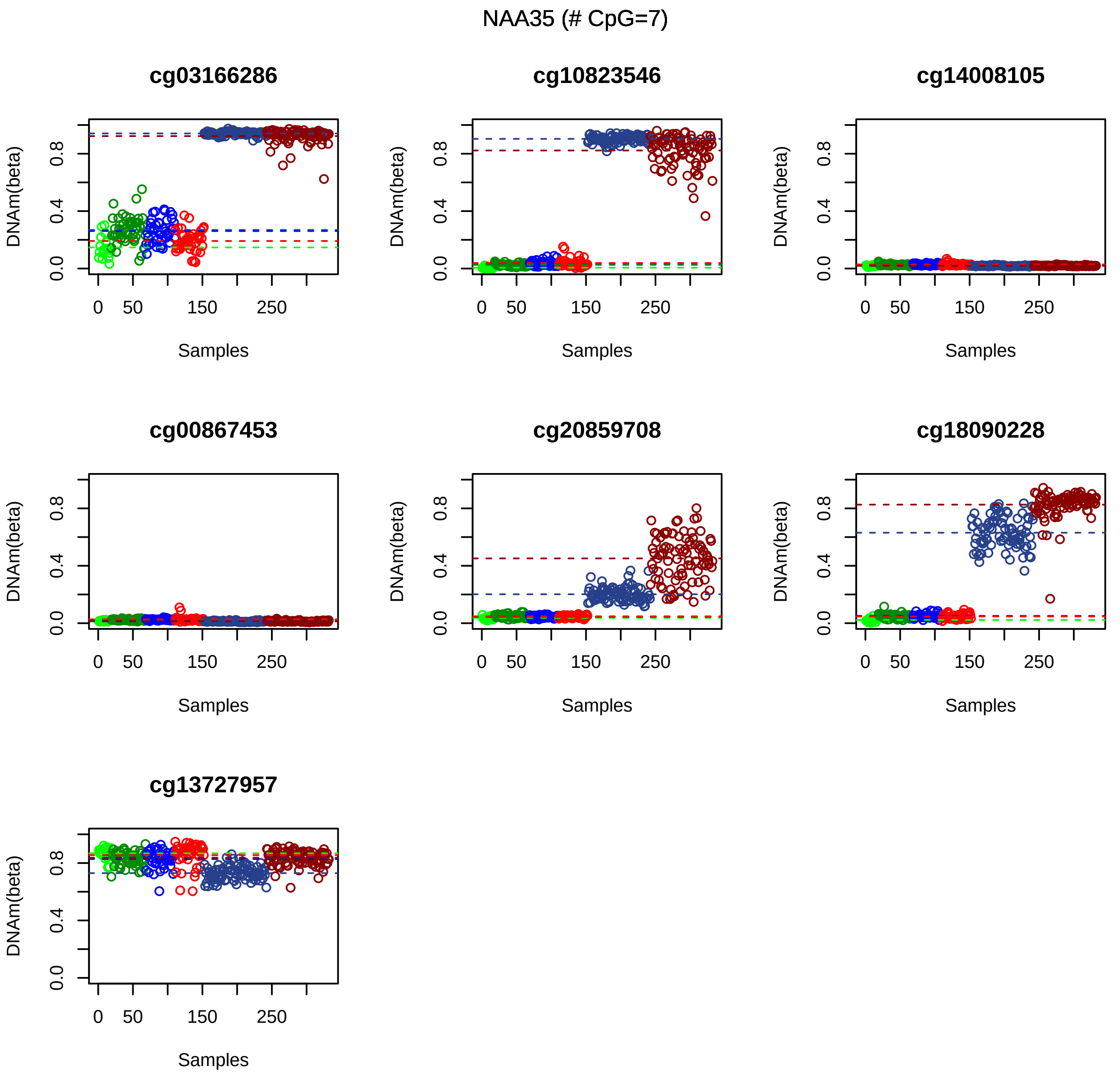
NAA35 (# CpG=7)
cg03166286
cg10823546
cg14008105
cg00867453
cg20859708
cg18090228
cg13727957
DNAm(beta)
0.0
0.4
0.8
Samples
0
50
150
250

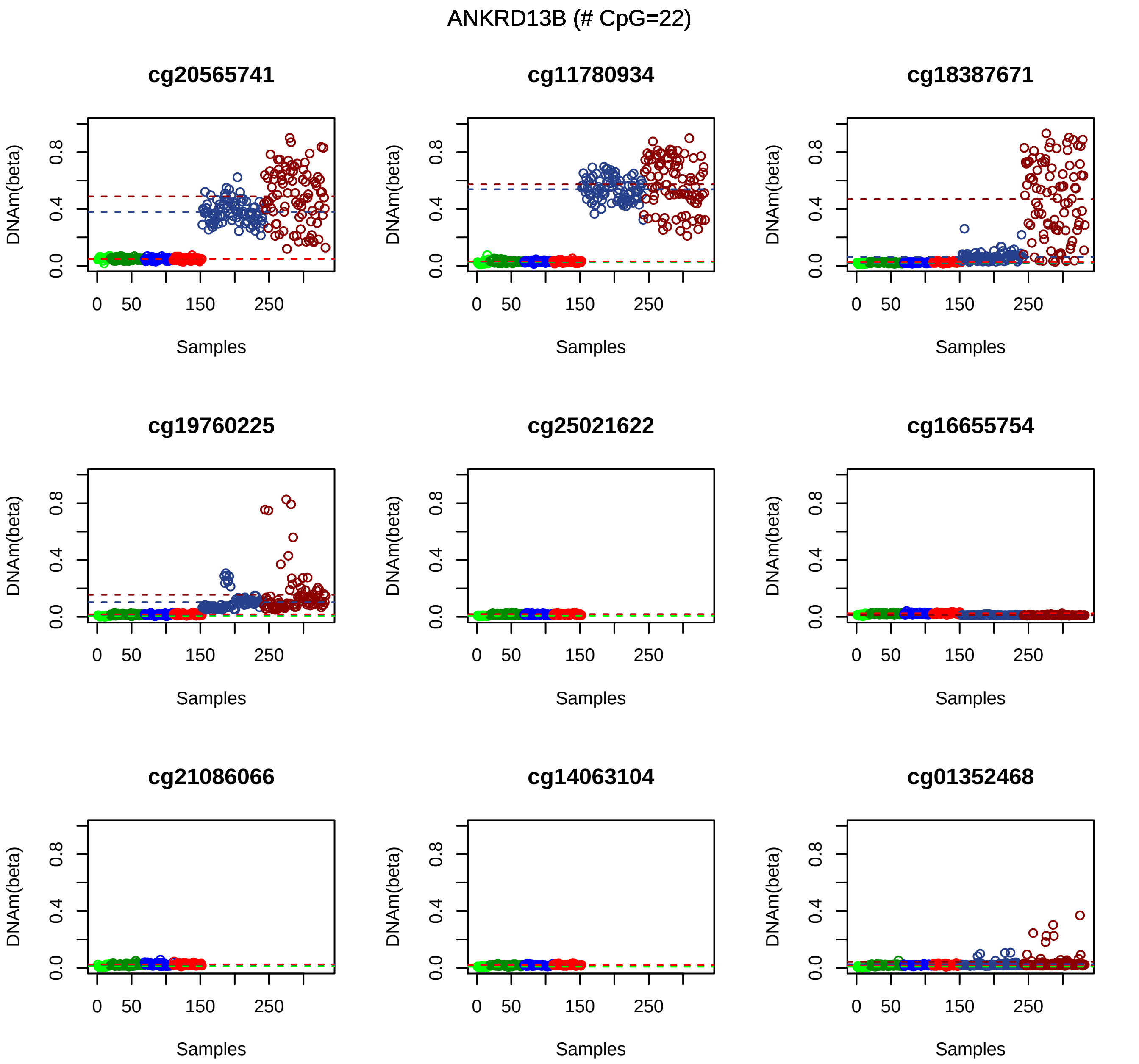
ANKRD13B (# CpG=22)
cg20565741
cg11780934
cg18387671
cg19760225
cg25021622
cg16655754
cg21086066
cg14063104
cg01352468
DNAm(beta)
0.0
0.4
0.8
Samples
0
50
150
250

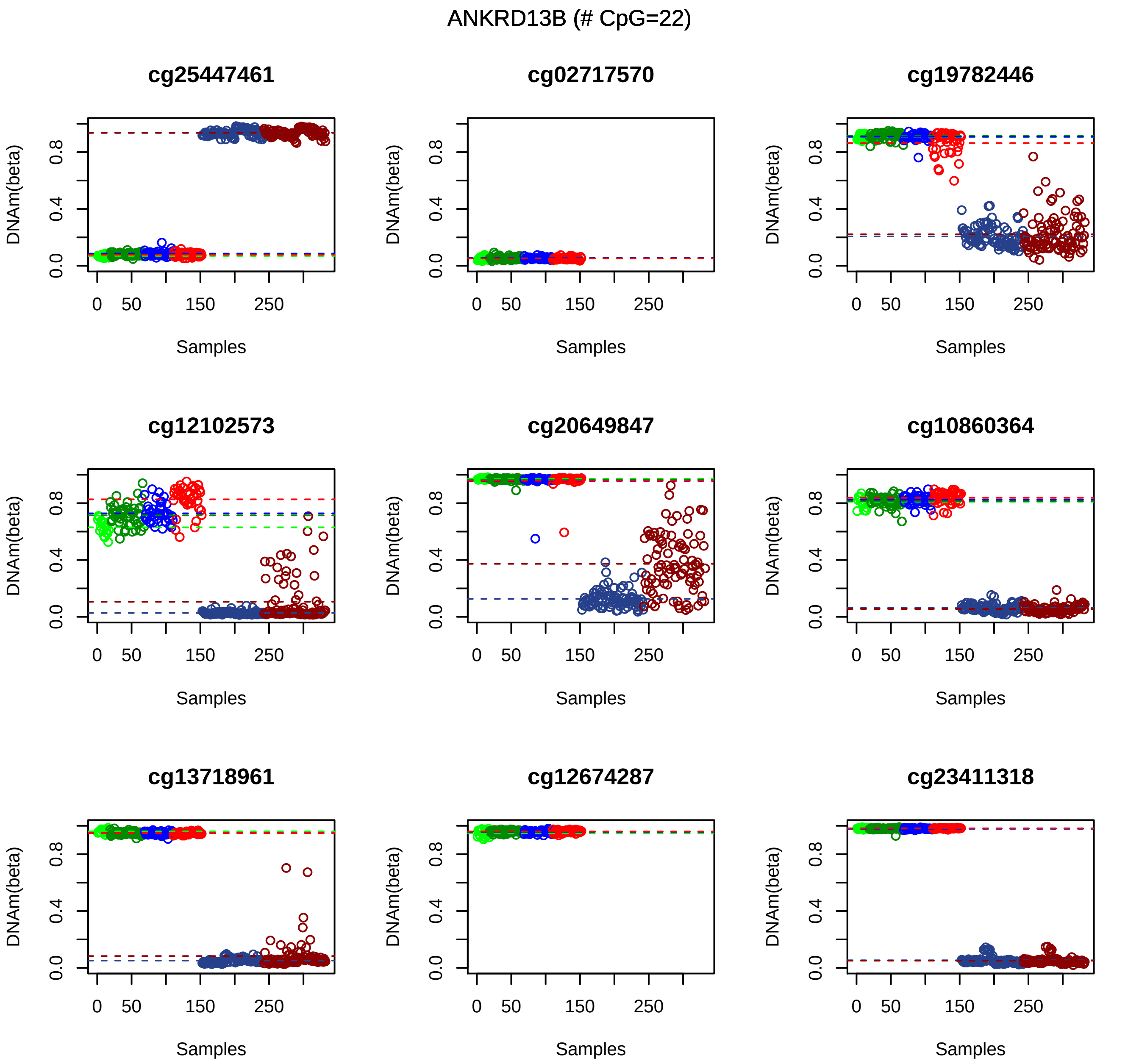

ANKRD13B (# CpG=22)
cg25447461
cg02717570
cg19782446
cg12102573
cg20649847
cg10860364
cg13718961
cg12674287
cg23411318
DNAm(beta)
0.0
0.4
0.8
Samples
0
50
150
250

ANKRD13B (# CpG=22)

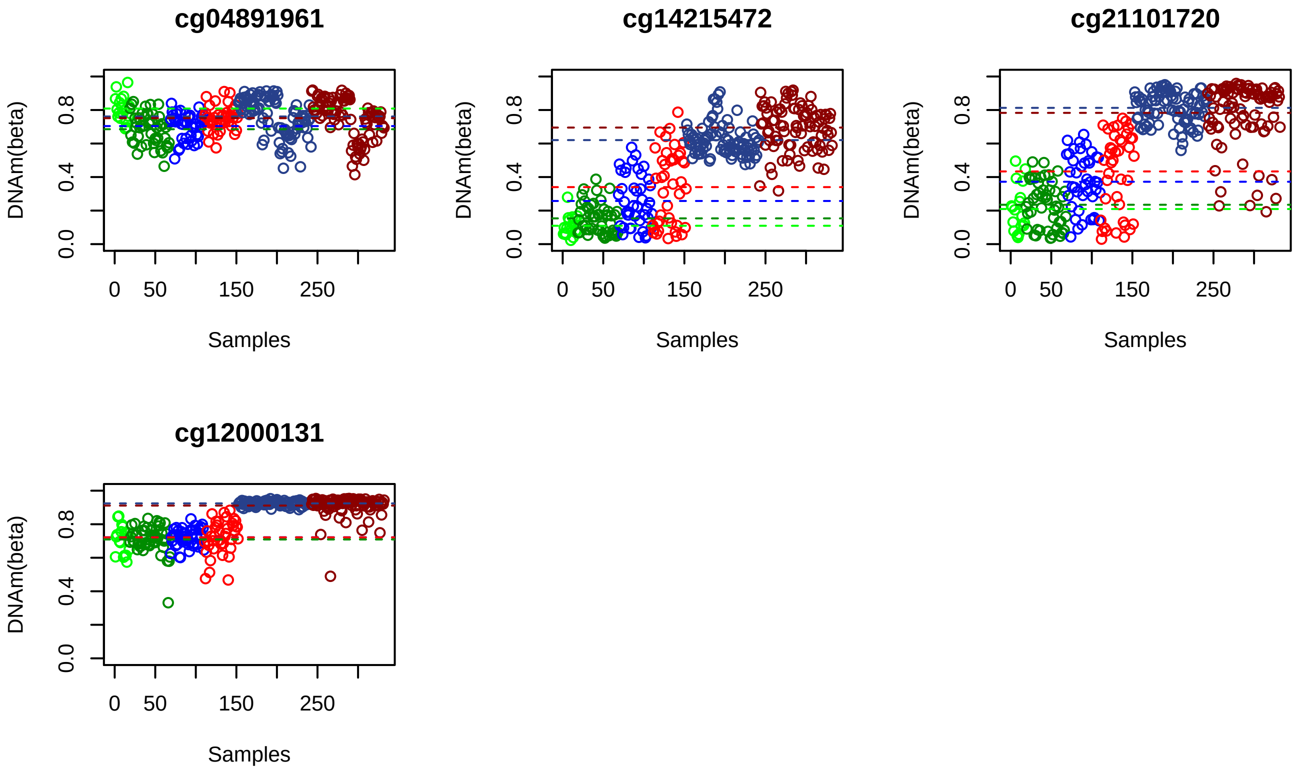

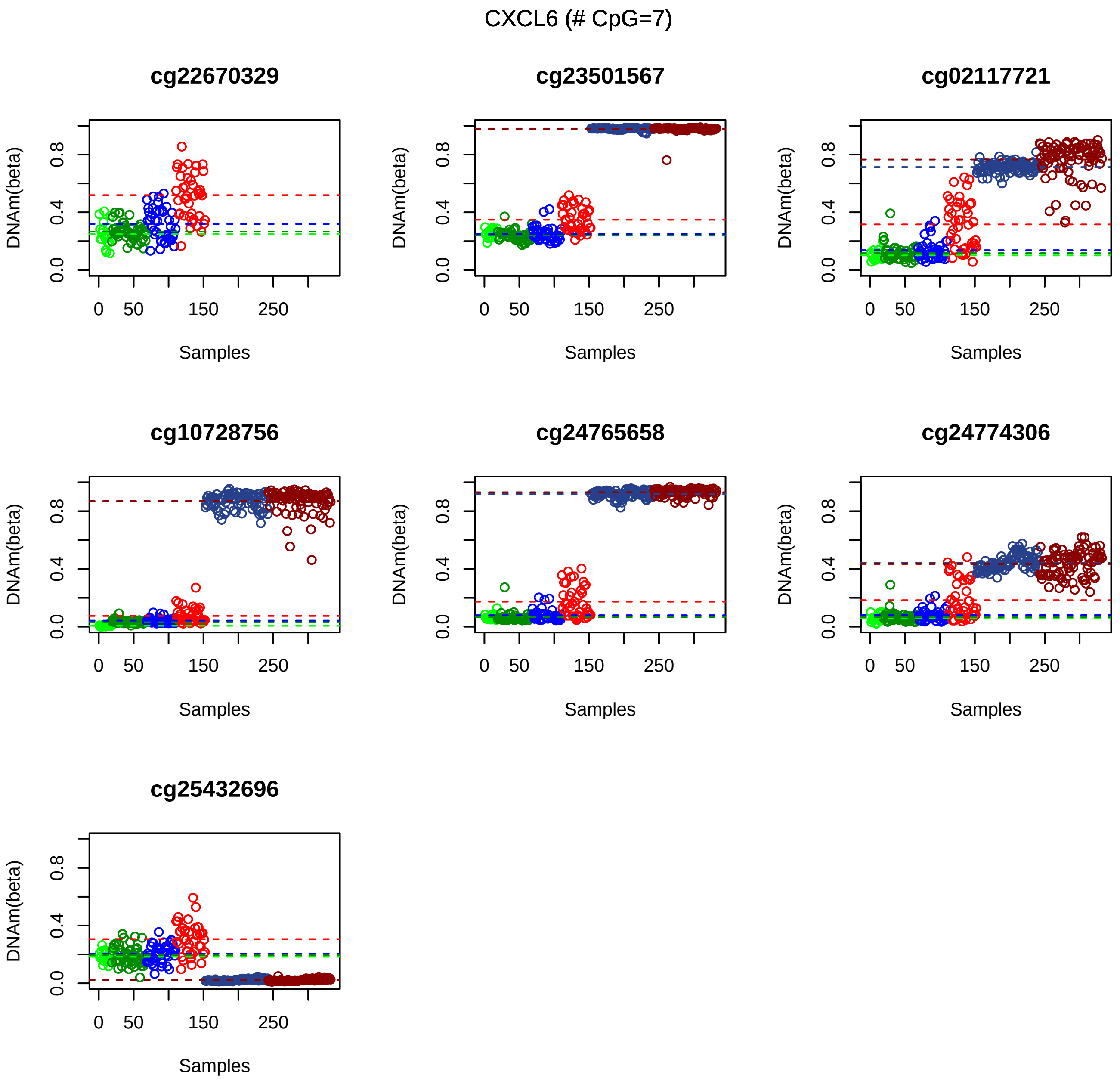
CXCL6 (# CpG=7)
cg22670329
cg23501567
cg02117721
cg10728756
cg24765658
cg24774306
cg25432696
DNAm(beta)
Samples
0.0
0.4
0.8
0
50
150
250

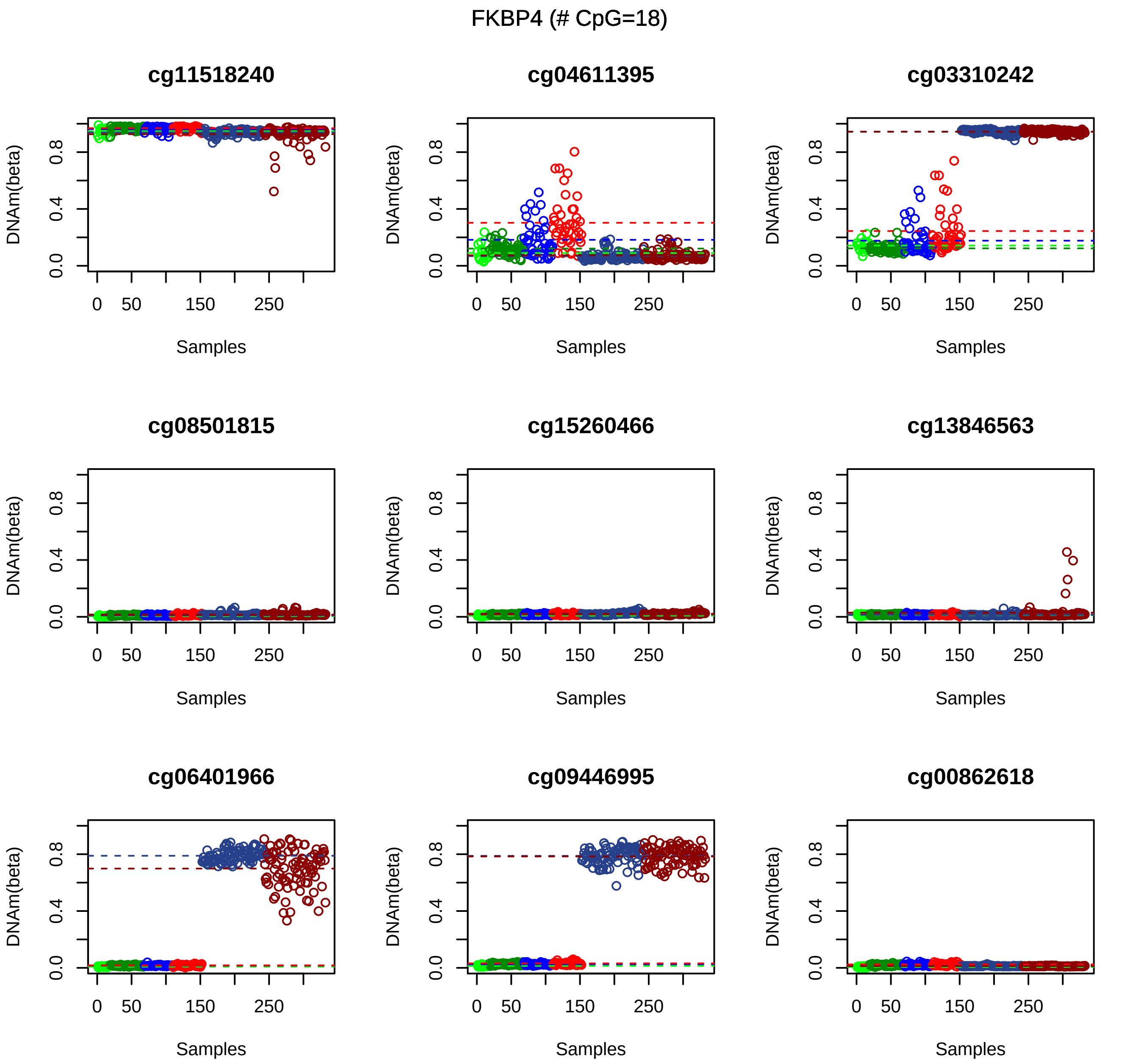

FKBP4 (# CpG=18)
cg11518240
cg04611395
cg03310242
cg08501815
cg15260466
cg13846563
cg06401966
cg09446995
cg00862618
DNAm(beta)
0.0
0.4
0.8
0
50
150
250
Samples

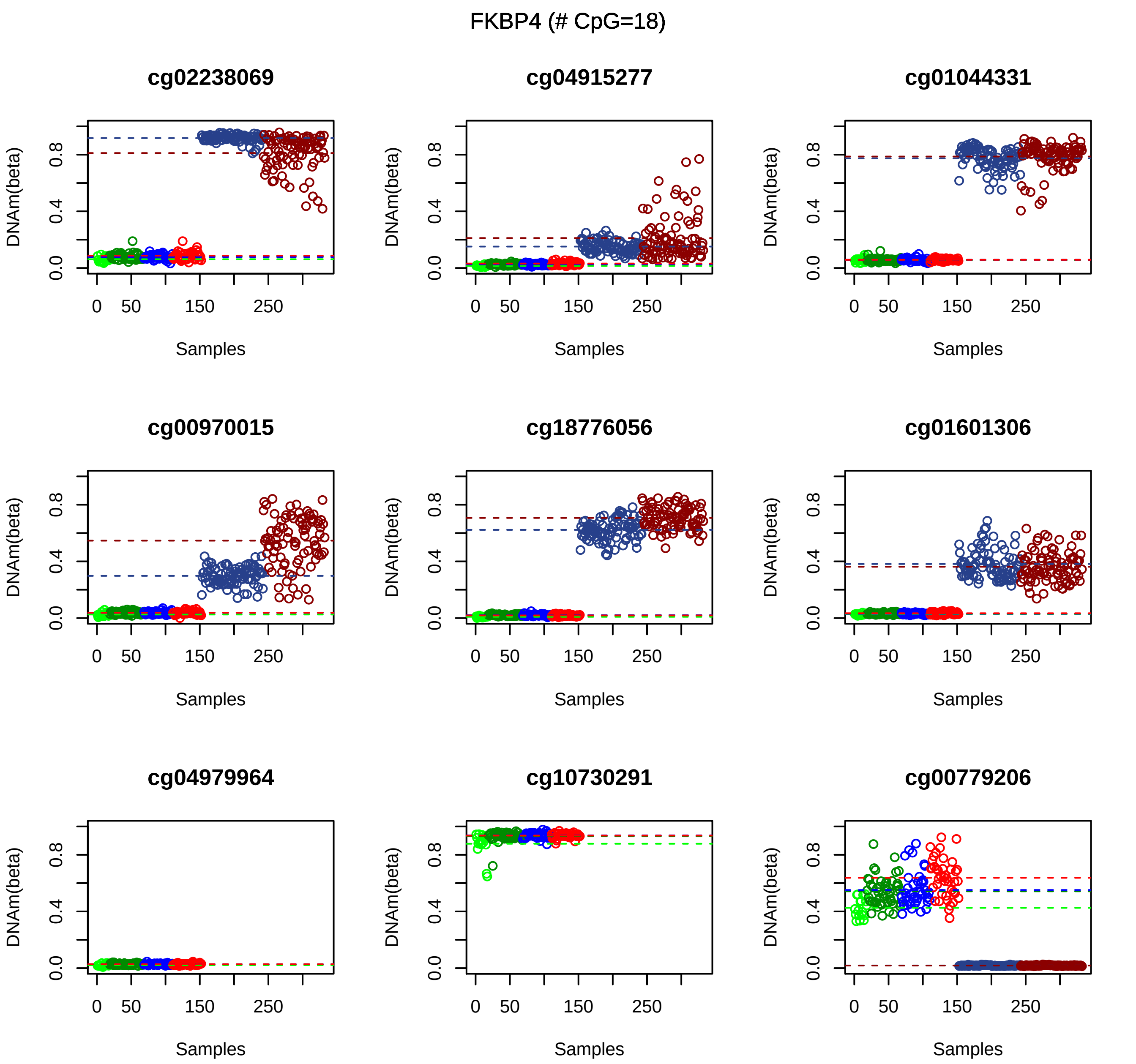

FKBP4 (# CpG=18)
cg02238069
cg04915277
cg01044331
cg00970015
cg18776056
cg01601306
cg04979964
cg10730291
cg00779206
DNAm(beta)
0.0
0.4
0.8
0
50
150
250
Samples

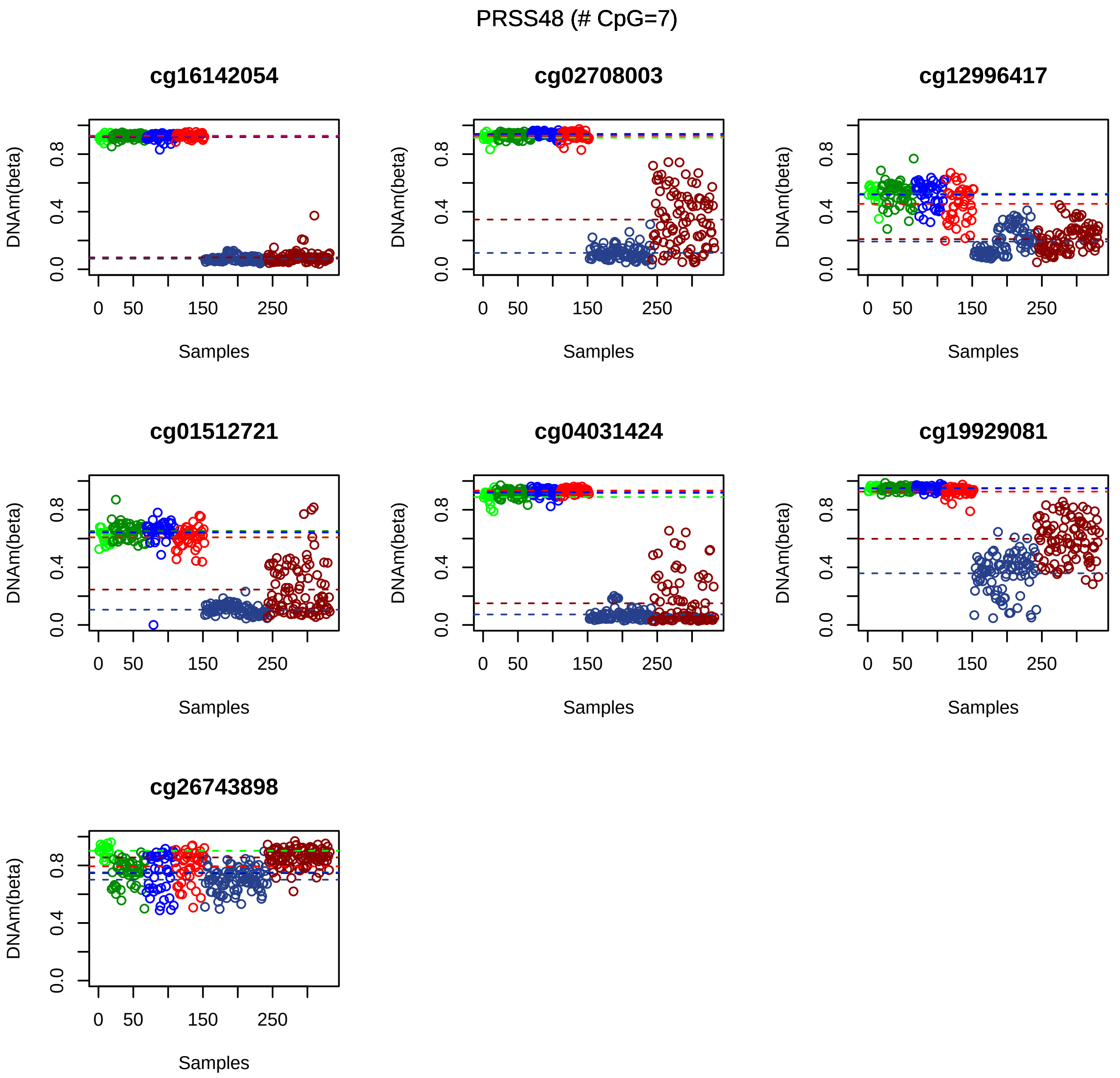
PRSS48 (# CpG=7)
cg16142054
cg02708003
cg12996417
cg01512721
cg04031424
cg19929081
cg26743898
DNAm(beta)
0.0
0.4
0.8
Samples
0
50
150
250

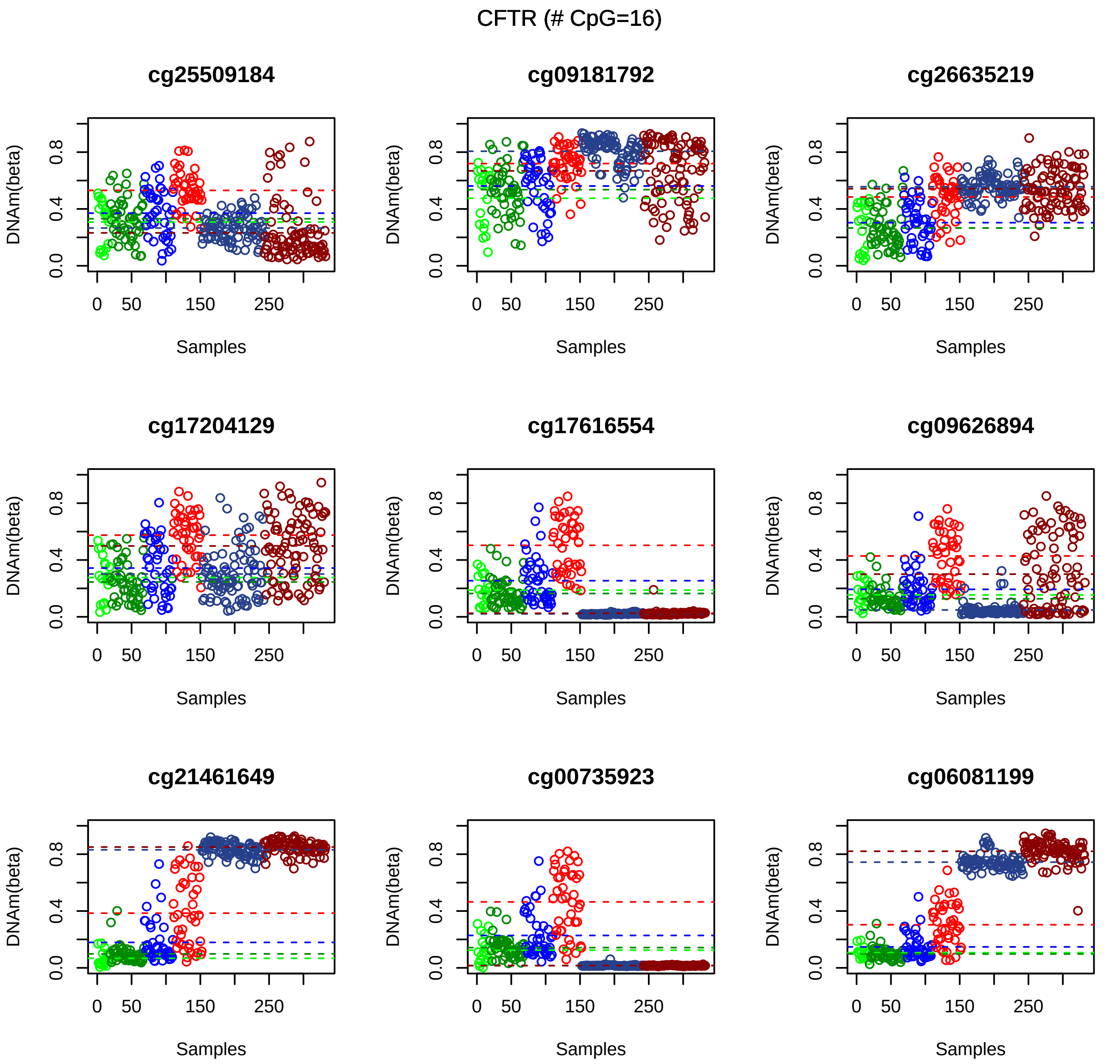
CFTR (# CpG=16)
cg25509184
cg09181792
cg26635219
cg17204129
cg17616554
cg09626894
cg21461649
cg00735923
cg06081199
DNAm(beta)
0.0
0.4
0.8
0
50
150
250
Samples

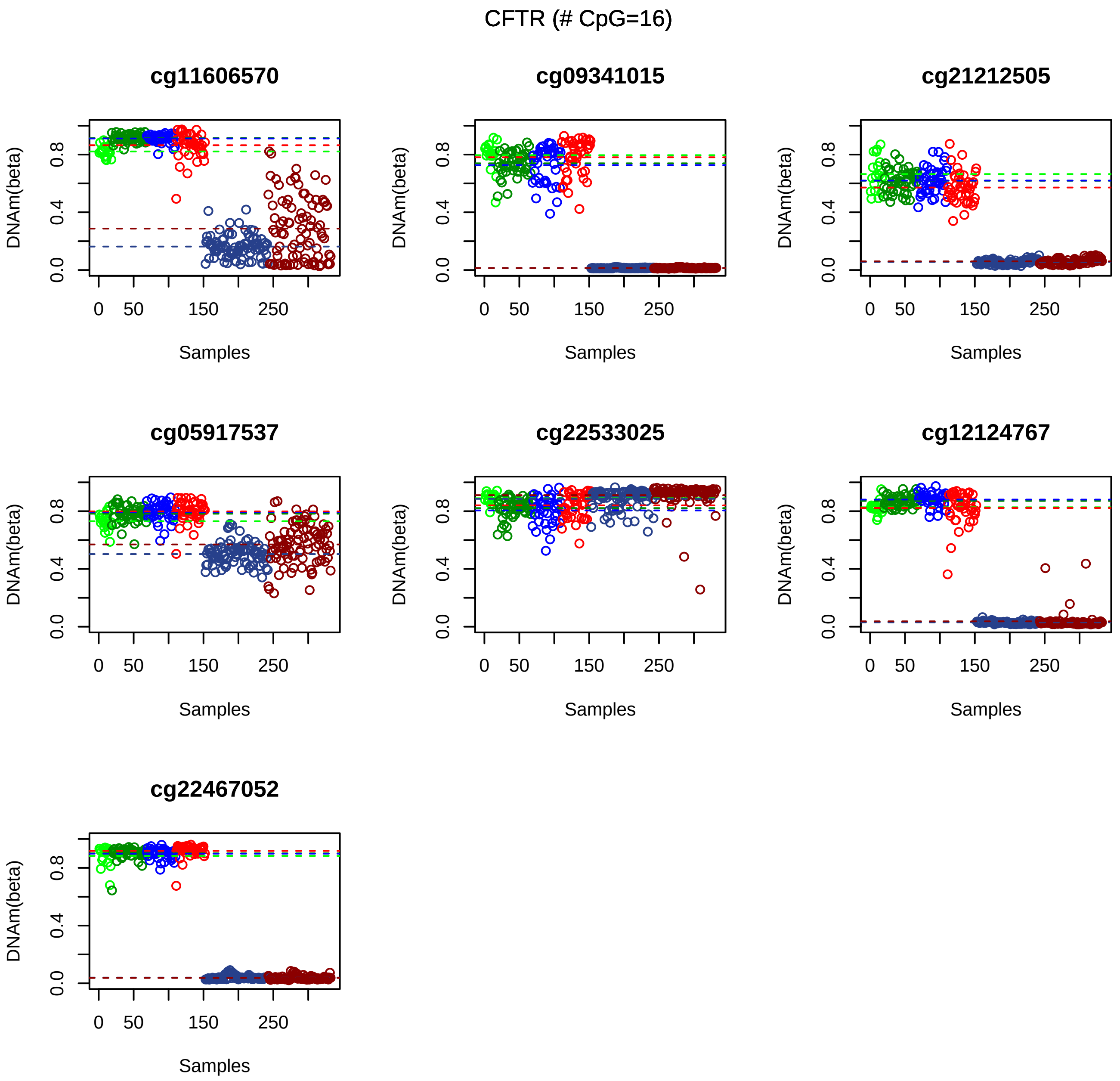

CFTR (# CpG=16)
cg11606570
cg09341015
cg21212505
cg05917537
cg22533025
cg12124767
cg22467052
DNAm(beta)
Samples
0.0
0.4
0.8
0
50
150
250
